# Serotype switching in *Pseudomonas aeruginosa* ST111 enhances adhesion and virulence

**DOI:** 10.1371/journal.ppat.1012221

**Published:** 2024-12-02

**Authors:** Mikkel Anbo, Mahbuba Akter Lubna, Dina A. Moustafa, Telmo O. Paiva, Laura Serioli, Kinga Zor, Claus Sternberg, Katy Jeannot, Oana Ciofu, Yves F. Dufrêne, Joanna B. Goldberg, Lars Jelsbak

**Affiliations:** 1 Department of Biotechnology and Biomedicine, Technical University of Denmark, Kgs. Lyngby, Denmark; 2 Department of Pediatrics, Division of Pulmonary, Asthma, Cystic Fibrosis, and Sleep, Emory University School of Medicine, Atlanta, Georgia, United States of America; 3 Louvain Institute of Biomolecular Science and Technology, UCLouvain, Louvain-la-Neuve, Belgium; 4 Center for Intelligent Drug Delivery and Sensing Using Microcontainers and Nanomechanics, Department of Health Technology, Technical University of Denmark, Kgs. Lyngby, Denmark; 5 Bioinnovation Institute Foundation, Copenhagen, Denmark; 6 Laboratory of Bacteriology, Associated Laboratory to French National Reference Center for Antibiotic Resistance, Teaching hospital of Besançon, France; 7 Costerton Biofilm Center, Department of Immunology and Microbiology, University of Copenhagen, Denmark; University of Maryland, UNITED STATES OF AMERICA

## Abstract

Evolution of the highly successful and multidrug resistant clone ST111 in *Pseudomonas aeruginosa* involves serotype switching from O-antigen O4 to O12. How expression of a different O-antigen serotype alters pathogen physiology to enable global dissemination of this high-risk clone-type is not understood. Here, we engineered isogenic laboratory and clinical *P*. *aeruginosa* strains that express the different O-antigen gene clusters to assess the correlation of structural differences of O4 and O12 O-antigens to pathogen-relevant phenotypic traits. We show that serotype O12 is associated with enhanced adhesion, type IV pili dependent twitching motility, and tolerance to host defense molecules and serum. Moreover, we find that serotype O4 is less virulent compared to O12 in an acute murine pneumonia infection in terms of both colonization and survival rate. Finally, we find that these O-antigen effects may be explained by specific biophysical properties of the serotype repeat unit found in O4 and O12, and by differences in membrane stability between O4 and O12 expressing cells. The results demonstrate that differences in O-antigen sugar composition can affect *P*. *aeruginosa* pathogenicity traits, and provide a better understanding of the potential selective advantages that underlie serotype switching and emergence of serotype O12 ST111.

## Author summary

Hospital-acquired infections caused by multidrug-resistant (MDR) or extensively drug-resistant (XDR) *Pseudomonas aeruginosa* are a growing concern, frequently linked to a small number of epidemic "high-risk clones" that have become prevalent in healthcare settings worldwide. These clones are particularly challenging to manage due to their enhanced resistance to antibiotics, making treatment options limited and less effective. In this study, we explore one factor that may contribute to the success and persistence of these clones: the O-antigen. The O-antigen is a long chain of sugars that extends from the bacterial surface, playing a key role in the organism’s interaction with its environment and in how it is recognized by the host’s immune system. Our research demonstrates that chemical variations in the different O-antigens found in *P*. *aeruginosa* can significantly influence how bacteria adhere to surfaces, modulate protein activity, and impact the progression of infection. These findings shed new light on the diverse biological functions of the O-antigen and help to explain why certain high-risk clones, such as ST111, have become globally successful. Understanding these mechanisms is crucial for developing new strategies to combat the spread of these highly resistant bacterial strains in healthcare environments.

## Introduction

The increasing prevalence of nosocomial infections caused by multidrug-resistant (MDR) or extensively drug-resistant (XDR) *Pseudomonas aeruginosa* is frequently linked to a limited number of so-called epidemic ‘high-risk clones´ (HiRiCs) that are widespread in hospitals worldwide [1,2]. HiRiCs are associated with poor clinical outcomes [1,2] and contain a large and diverse number of horizontally acquired resistance determinants [3], yet the molecular mechanisms that enable particular clone types (but not others) to emerge as HiRiCs remain poorly understood.

We have previously shown that evolution of one of the most globally disseminated HiRiCs, ST111 serotype O12, is associated with a serotype switch in which the native O-antigen O4 biosynthesis gene cluster was replaced by the O12 gene cluster by horizontal gene transfer and recombination [4]. Although ST111 is globally associated both with serotype O12 and O4, ST111 O12 is by far more prevalent than ST111 O4 among antibiotic resistant clinical isolates [5–8], suggesting that the switch from O4 to O12 expression provides ST111 with unknown fitness advantages. In support of this, we have also observed multiple, independent serotype switches from O4 to O12 in ST111 isolates across different continents [4,9], and thus it appears that there is a strong selection for the recombinatorial replacement of O4 with O12 in ST111. Other observations also point towards an important role of serotype O12 in additional clone types. For example, we have previously observed—albeit sporadically–serotype switches from other serotypes to O12 in other clone types, such as ST253 (PA14-like, usually associated with serotype O19) and ST244 (usually associated with serotype O2/O5) [9]. Furthermore, serotype O12 strains have been associated with multidrug resistance in Europe since the 1980s [10]. The aim of the present study was to determine how the switch from serotype O4 to O12 alters pathogen behavior, fitness or virulence of ST111 to explain the selective advantage of the serotype switch and the global success of this devastating clone type.

The lipopolysaccharide (LPS) is a major virulence factor of *P*. *aeruginosa* which has been shown to interact with myriads of molecular species, including host molecules, antibiotics, and bacteriophages [11–16]. LPS is composed of the lipid A, a core polysaccharide, and a variable O-specific antigen (OSA) or the common polysaccharide antigen (CPA). The O-antigen is composed of repeat units of 2–5 sugars and their chemical structures, and the underlying genetics have been described previously [12,17]. The variable and diverse structures of repeating sugar subunits in OSA classify *P*. *aeruginosa* into 20 distinct serotypes [18–20]. By the disruption of O-antigen synthesis through genetic knockouts, many studies have previously documented the importance of the O-antigen to clinically relevant phenotypes such as virulence in models of acute infection [21], antibiotic susceptibility [15,16,22], and host complement-mediated killing [23]. Although these findings clearly illustrate the general importance of the O-antigen structure, we currently do not understand how the different sugar compositions found in the various O-antigens expressed on *P*. *aeruginosa* affect the biology of the pathogen. The serotype switch documented in ST111 strongly suggests that different O-antigen structures may indeed have different, yet unknown, effects on *P*. *aeruginosa* biology and virulence. Similarly, studies have shown that a number of serotypes predominate in clinical settings and epidemic outbreaks [24], suggesting a link between serotypes and pathogen behavior and physiology. However, these epidemiological studies do not offer straightforward explanations as to why particular serotypes would predominate and how different serotypes affect pathogen biology.

Associating particular phenotypes to specific O-antigen structures by comparing clinical isolates of different serotypes is not straightforward and confounded by the genetic diversity of the different *P*. *aeruginosa* hosts. To overcome this problem, we constructed isogenic strains that express either O4 or O12 serotype with the goal of defining how these different O-antigen structures affect *P*. *aeruginosa* physiology. Here, we show that adhesion properties and virulence phenotypes are differentially affected by heterologous expression of O4 and O12 in different *P*. *aeruginosa* backgrounds, specifically we noted that O12 is more adherent and virulent in our models. We furthermore identify serotype-specific alterations in membrane stability and function of membrane associated proteins as the mechanism underlying the adhesion and virulence phenotypes. These results may help to explain the global clonal success of multi-drug resistant ST111 serotype O12.

## Materials and methods

### Ethics statement

All animal procedures were conducted according to the guidelines of the Emory University Institutional Animal Care and Use Committee (IACUC), under approved protocol number PROTO 201700441. The study was carried out in strict accordance with established guidelines and policies at Emory University School of Medicine, and recommendations in the Guide for Care and Use of Laboratory Animals, as well as local, state, and federal laws.

### Bacterial strains, plasmids and culture conditions

Bacterial strains and plasmids used in this study are described in Tables 1 and 2, respectively. *P*. *aeruginosa* and *E*. *coli* overnight cultures were routinely grown in LB for 16–18 hours at 37°C, 200 rpm unless stated otherwise. Cells were stored at -80°C in a 20% glycerol solution and streaked on LB agar (2%) for further sub-culturing. For selection or maintenance of plasmids in *E*. *coli* the following supplements were added: 8 μg/ml tetracycline, 100 μg/ml ampicillin, 35 μg/ml kanamycin, 10 μg/ml gentamycin, 6 μg/ml chloramphenicol. For selection of *P*. *aeruginosa* the following supplements were added: 50–100 μg/ml tetracycline, 50 μg/ml gentamycin. Competent *E*. *coli* cells were prepared by repeat washing with 100mM CaCl_2_ + 15% glycerol and 50 μl aliquots were transformed by addition of plasmid and heat shock (30s at 42°C, 2 min on ice, rescued for 1–2 h at 37°C after addition of 1 ml LB). Expression of O-antigen was verified by agglutination with *Pseudomonas aeruginosa* monovalent anti-serum P4, P5, P10 and P12 (Bio-Rad).

**Table 1:** Bacterial strains used in this study. *Abbreviations used: ETγA for genes recE, recT, redγ, and recA. OSA for O-specific antigen.

Name	Description*	Source
***E*. *coli* strains**		
Cc118λpir	Plasmid propagation	[25]
GBdir-pir116	Arabinose inducible ETγA operon	[26]
***P*. *aeruginosa* strains**		
PAO1	Laboratory strain	ATCC15692
PA14	Laboratory strain	Janus Haagesen, DTU Biosustain
ST111 2875	Strain 2875, serotype O4	[27]
ST111 2879	Strain 2879, serotype O12	[27]
IATS O4	Serotype O4 type strain	Joseph Lam
IATS O12	Serotype O12 type strain	Joseph Lam
PAO1ΔO	PAO1 Δ*wbpM*1741-3547432 (deletes OSA)	This study
PA14ΔO	PA14 Δ2026751-*wbpM*1741 (deletes OSA)	This study
ST111ΔO	ST111 2875 Δ2113766-*wbpM*1741 (deletes OSA)	This study
PA14ΔO+O4	PA14 ΔO attTn*7*::O4	This study
PA14ΔO+O12	PA14 ΔO attTn*7*::O12	This study
PA14ΔO+O19	PA14 ΔO attTn*7*::O19	This study
PAO1ΔO+O4	PAO1 ΔO attTn*7*::O4	This study
PAO1ΔO+O12	PAO1 ΔO attTn*7*::O12	This study
PAO1ΔO+O5	PAO1 ΔO attTn*7*::O5	This study
ST111ΔO+O4	ST111 ΔO attTn*7*::O4	This study
ST111ΔO+O12	ST111 ΔO attTn*7*::O12	This study
PAO1 galU	Rough LPS mutant	[28]
PAO1pilA	Type IV pili deficient mutant	[29]

**Table 2:** Plasmids used in this study. *Abbreviations and nomenclature: OSA for O-specific antigen. eYFP for enhanced yellow fluorescent protein. msfGFP for monomeric superfolder green fluorescent protein. mKate is a red fluorescent protein. CamR for chloramphenicol resistant. AmpR for ampicillin resistant. SacB is the Bacillus subtilis levansucrase gene. TetR for tetracycline resistant. KmR for kanamycin resistant, and GmR for gentamycin resistant.

Name	Description*	Selection	Source
pRK600	Conjugation helper plasmid	CamR	[30]
pTNS2	Tn*7* helper plasmid	AmpR	[31]
pFLP3	Flp expression vector	SacB AmpR TetR	[31]
pNJ1	Allelic replacement	TetR SacB	[32]
pMA03 PAO1	pNJ1 derivate, facilitates deletion of OSA cluster in PAO1	TetR SacB	This study
pMA03 PA14	pNJ1 derivate, facilitates deletion of OSA cluster in PA14	TetR SacB	This study
pMA03 ST111	pNJ1 derivate, facilitates deletion of OSA cluster in ST111	TetR SacB	This study
pMA10 PAO1	pNJ1 derivate, facilitates integration of *tetA* in serotype cluster of PAO1	TetR SacB	This study
pMA10 PA14	pNJ1 derivate, facilitates integration of *tetA* in serotype cluster of PA14	TetR SacB	This study
pMA10 O4	pNJ1 derivate, facilitates integration of *tetA* in serotype cluster of IATS O4	TetR SacB	This study
pMA10 O12	pNJ1 derivate, facilitates integration of *tetA* in serotype cluster of IATS O12	TetR SacB	This study
pUC18R6kT-mini-Tn7T-Km	Tn*7* tagging vector with R6K oriR incP oriT	AmpR, KmR	[31]
Mini-tn7(GM) PA_A1/04/03_-eyfp-A	Tn*7* tagging vector with eYFP	GmR, CamR, AmpR, eYFP	[33]
pMA09	Mini-tn*7* derivate, with eYFP integrated between two transcriptional terminators	AmpR, KmR, eYFP	This study
pMA11 O5	pMA09 derivate, with PAO1 serotype cluster (O5) replacing eYFP	TetR, KmR, AmpR, SacB	This study
pMA11 O19	pMA09 derivate, with PA14 serotype cluster (O19) replacing eYFP	TetR, KmR, AmpR, SacB	This study
pMA11 O4	pMA09 derivate, with IATS O4 serotype cluster (O4) replacing eYFP	TetR, KmR, AmpR, SacB	This study
pMA11 O12	pMA09 derivate, with IATS O12 serotype cluster replacing eYFP	TetR, KmR, AmpR, SacB	This study
pSEVA658::pilA_PA14_	pSEVA658 overexpression vector for pilA (from PA14), inducible by addition of m-toluate	Gm	This study

### Bacterial growth media

For bacterial growth, the following culture media were used: Lysogeny broth (LB; Carl Roth, X964), Pseudomonas isolation agar (PIA; Millipore, 17208), Low salt LB (LSLB; 10 g/l tryptone, 5 g/l yeast extract, 0.5 g/l NaCl) and no salt LB agar (NSLB; 10 g/l tryptone, 5 g/l yeast extract, 15 g/l agar, 6% sucrose). For solid growth media, agar (ITW Reagents, A0949) was supplemented at 2% (w/v) unless otherwise stated. Cultures were regularly washed with PBS (8 g/l NaCl, 0.2 g/l KCl, 1.44 g/l Na_2_HPO_4_·2H_2_O, 0.2g/l KH_2_PO_4_).

### DNA manipulation

Genomic DNA purification for use in cloning or whole-genome sequencing was purified using the Monarch HMW DNA Extraction Kit for Tissue (T3060L, New England Biolabs) according to manufacturer’s instruction with one modification: 1 ml overnight culture was washed with 1 ml PBS prior to first step. Plasmid DNA was purified using the Macherey Nagel Plasmid purification kit, according to manufacturer’s specifications. For cloning, DNA was routinely purified with the NucleoSpin gel and PCR clean-up kit (Macherey-Nagel) according to the manufacturer’s instruction or by ethanol precipitation, and verified by gel electrophoresis. DNA was quantified by gel electrophoresis or with a Qubit fluorometer (Thermofisher).

### Allelic replacement for gene deletion and genomic integration

Gene deletions or integrations were carried out according to Hmelo et al [34] using the allelic replacement vector pNJ1 [32]. Briefly for construction of gene deletions, regions up- and downstream of target gene(s) were PCR amplified and joined by SOE PCR (specifics about methods can be found in S1 Supplementary Methods and S3 Table). For deletion of the OSA cluster, the deletion construct fragments were 5.6, 5.6, and 5.3 kb for PA14, ST111 and PAO1, respectively. Constructs were ligated to pNJ1 after restriction digestion and used for transformation of calcium competent *E*. *coli*. These were then transferred to *P*. *aeruginosa* by conjugation by addition of helper strain containing pRK600. Briefly for genomic integration, a target region was PCR amplified and ligated to pNJ1 after restriction digestion.

### Direct cloning of serotype clusters in *P*. *aeruginosa*

RecET mediated recombineering was carried out according to Wang et al [26] using the pR6kT-mini-Tn7T-Km [31] vector to capture and transfer serotype gene clusters to *P*. *aeruginosa* with the following modifications: the donor DNA was tagged by integration of pNJ1 in the serotype cluster, to provide a selection for successful genomic capture. A detailed description of the individual steps can be found in S1 Supplementary Methods and primers in S3 Table. Transfer of constructs to attTn*7* in *P*. *aeruginosa* was done according to Choi et al [31] by four-parental mating and verified by PCR. Selection markers, including the pNJ1 plasmid, were removed by sucrose counter-selection and subsequent flp/frt recombination.

### Direct cloning of fluorescent markers to mini-ctx2

PCR fragments from pBG42 [35] and pBG42 mKate were designed with overhangs homologous to mini-ctx2 [36] using PCR. These fragments contain *aacC1* (gentamycin resistance) and msfGFP or mKate. Plasmid preparation of mini-ctx2 was digested using SacI (Thermofisher) and purified. RecET recombineering was used to construct mini-ctx2 *aacC1* msfGFP and mini-ctx2 *aacC1* mKate. These plasmids integrate into the CTX attachment site in the *P*. *aeruginosa* genome and thus mediate genomic tagging of *P*. *aeruginosa* with *aacC1* and a green or red fluorescent marker. The plasmids were introduced into *P*. *aeruginosa* by conjugation according to Hoang et al [36].

### Whole-genome sequencing and assembly

Illumina sequencing was carried out on the Novaseq 6000 platform using 2x150 bp paired-end reads. Nanopore sequencing was barcoded using the rapid barcoding kit (SQK-RBK004) and sequenced on a R9.4.1 flowcell using the MinION device. Nanopore reads were basecalled using MinKNOW (3.1.19). FastP (0.12.4) [37] was using for quality control of Illumina reads, including adapter trimming and base correction (flag–c) for paired-end data. Reads were subset by seqtk (1.3-r106) [38] with random seeds for paired-end Illumina reads. For long-read sequencing, porechop (0.2.4) [39] was used for read demultiplexing and adapter trimming and reads were subset using filtlong (0.2.0) [40] with–min_length 1000. Hybrid and Illumina-only assemblies were performed using Unicycler (0.5.0) [41] and these were annotated by the prokaryotic genome annotation pipeline (PGAP) (v6.5) [42].

### Adhesion of *P*. *aeruginosa* strains

Bacterial biofilm formation in tissue culture plates was quantified according to protocols of O’Toole & Kolter [43] using crystal violet staining, to focus on the initial attachment of bacteria. Briefly, overnight cultures, grown in LB at 37 for up to 18 hours, were washed 2x in PBS by centrifugation at 5000*g* for 3 min. The OD_600_ of each culture was adjusted to OD_600_ = 1 in PBS and 200 μl culture was transferred to wells of a microtiter plate. 200 μl PBS was added to empty wells and used as blanks. Microtiter plates were incubated for 2 hours at 37°C with lid on, then washed 3x by flicking out liquid, and adding 250 μl distilled water to each well. Biofilms were stained by addition of 200 μl 0.1% crystal violet (freshly prepared on each day, dissolved in distilled water) for 15 minutes at room temperature. Plates were then washed 4x by flicking out liquid and adding 250 μl distilled water. 200 μl 96% ethanol was added to each well and the plate was incubated at room temperature for 30 minutes before measuring the 570nm absorbance of each well with a Biotek Cytation 5 or Synergy H1 plate-reader. Data was processed by an in-house script where the absorbance of blanks was subtracted from all absorbance values.

### Simulated partition coefficient of serotype repeat units

Chemical structures of the serotype repeat-units was characterized using ACD/ChemSketch 2014 using the addon logP (v.14.03) [44]. Briefly, the known structures of the serotype repeat units (O4, O5, O12, O19) were re-created in ChemSketch according to [12,17]. Using the logP addon, the relative polarity (logP) of each serotype structure could be estimated.

### Lysis rate of bacteria in EDTA+Lysozyme solution

*P*. *aeruginosa* strains were lysed in EDTA and lysozyme according to Ayres et al [45]. Briefly, bacterial cultures were grown overnight for 16–18 hours in LB broth at 37°C, 200 rpm. 1.5 ml culture was transferred to an Eppendorf tube and washed 3x by centrifugation at 5000*g* for 3 minutes in 1 ml 20 mM tris buffer (pH 7.8). The OD and volume of each culture was then adjusted to 1.2 ml OD = 1.25 in 20 mM tris buffer. A dilution row of EDTA was prepared in a microtiter plate using a multi-channel pipette. Lysozyme and bacterial suspension was added for a final concentration of 10 μg/ml and OD = 0.5, respectively, with a total volume of 250 μl in each well. After addition of bacterial suspension, the absorbance at 600nm was continuously read (at the lowest possible interval) with a Biotek Cytation 5 or Synergy H1 plate-reader at 30°C for 1 hour. The influence of magnesium was assayed in a similar way, but with a final concentration of 10mM MgSO_4_ in each well. The lysis rate in each well was determined from the kinetic curves using an in-house R script.

### Twitching motility

Twitching motility of *P*. *aeruginosa* strains was assayed according to Turnbull et al [46] on LB (1% Agar) plates. Briefly, a single colony was carefully touched with a 10μl pipette tip and stabbed through the agar layer to inoculate the petri dish plastic. Plates were inverted and stacked in a plastic box (3 plates/stack) and incubated at 37°C with the lid slightly ajar for 24 hours. An open petri dish containing a wet paper towel was placed inside the plastic box for humidity. Two perpendicular diameters were measured to quantify twitching motility of each assay, which was used to calculate the twitching motility area (*A* = *R*1 * *R*2 * *π*). To photograph twitching motility, the agar layer was carefully removed and the petri dish was photographed with a T:Genius gel imager using the UV transilluminator.

### Single cell atomic force spectroscopy

For single cell force spectroscopy experiments, bacterial probes were brought into contact with glass or polystyrene substrates [47,48]. Briefly, bacterial cultures were grown overnight for 16–18 hours in LB broth at 37°C, 200 rpm. These were washed twice in PBS by centrifugation and diluted 100-fold. Bacterial probes were subsequently prepared as previously described [49] by attaching single bacterial cells to hydrophobic AFM colloidal probes. AFM experiments were carried out in PBS at room temperature, using a JPK NanoWizard 4 NanoScience instrument. Force-distances curves were recorded while approaching and retracting bacterial probes to and from the substrates on areas of 10 μm x 10 μm, using a constant approach and retraction speed of 3 μm/s, a ramp length of 3 μm and an applied force setpoint of 0.25 nN, at a resolution of 16 x 16 pixels. The adhesion properties of each bacterial strain were characterized by measuring the magnitude and frequency of force plateaus in the retraction curves [47,50].

### Murine infection models

Mouse pneumonia infection was performed with six -to eight-week-old female BALB/c mice (Jackson Laboratories, Bar Harbor, ME) as previously described with a few modifications [51]. Briefly, mice were anesthetized by intraperitoneal injection of 0.2 ml of a cocktail of ketamine (10mg/ml) and xylazine (5 mg/ml). *P*. *aeruginosa* strains: PAO1, IATS O4, IATS O12, ST111, ST111-2, PAO1ΔO+O4 and PAO1ΔO+O12 were grown on Difco Pseudomonas Isolation Agar (PIA) for 16–18 hours at 37°C and suspended in PBS to an O.D_600_ of 0.5, corresponding to ~10^9^ CFU/ml and inocula were adjusted to obtain the desired challenge dose.

For acute pneumonia model, mice were intranasally instilled with indicates *P*. *aeruginosa* doses in 25 μl of PBS. For systemic infection, mice were infected by intraperitoneal injection of desired dose in 0.1 ml of P. aeruginosa strains as indicated. To assess the bacterial burden, mice were euthanized at 24 h post-infection and whole lungs were collected aseptically, weighed, and homogenized in 1 ml of PBS. Tissue homogenates were serially diluted and plated on PIA for CFU enumeration. For survival studies, infected mice were monitored up to 3 weeks post-infection. Animals that succumbed to infection or appeared to be under acute distress were humanely euthanized and were included in the experiment results.

### LPS purification & visualization

LPS was purified from 1 ml *P*. *aeruginosa* overnight cultures grown on LB agar that were normalized to OD_600_ = 0.5 (~1e9 CFU / ml). Cultures were pelleted by centrifugation and resuspended in 200 μl SDS sample buffer (BioRad) and briefly boiled. Samples were incubated with DNaseI (240 μg/ml), RNase (240 μg/ml) for 30 minutes at 37°C, followed by 3 hours incubation with Proteinase K (465 μg/ml) at 59°C. 10 μl purified LPS was loaded and separated on a 4–12% gradient Tris-glycine (BioRad) using SDS-PAGE and visualized by Western blotting or with the Pro-Q emerald lipopolysaccharide stain kit (Thermo Fischer) according to the manufacturer’s specifications. *P*. *aeruginosa* serogroup specific (O4, O12, O10/O19) rabbit polyclonal antibodies (Denka Seiken, Tokyo Japan) or *P*. *aeruginosa* anti O5 mouse IgM MF15-4 monoclonal antibody (MediMabs, cat # MMM-76605-1) were used for Western blotting. Anti-mouse IgM-HRP or anti-rabbit IgG-HRP were used as secondary antibodies, respectively.

### Serum bactericidal assay

Overnight cultures were diluted in PBS supplemented with 1% proteose peptone to OD_600_ = 0.05 for a final inoculum of approximately 1e6 CFU per well. Normal human serum (NHS) was diluted in PBS plus 1% proteose peptone to twice the final concentration. As controls, cultures were additionally incubated with NHS that was heat inactivated (HI) by incubation at 56°C for 30 minutes, or PBS +1% proteose peptone (0% serum). Equal volumes (50 μl) of sera and bacterial suspensions were mixed and incubated at 37°C for 1 hour with gentle shaking. An aliquot from each well was serially diluted and plated on LB agar for CFU enumeration after incubation overnight at 37°C. Serum survival was determined for each strain by determining the log10 fold difference between CFU recovered after incubation with NHS and HI.

### Statistical tests and data analysis

Rstudio (2022.07.2 build 576) was used for statistical testing. For comparison of multiple means, the compare_means function of ggpubr [52] was used with method = “t.test” and correction for multiple testing with false discovery rate (FDR). For comparisons of means between two means, the built-in function t.test from R stats was used (Welch’s t-test). The results of animal survival studies are presented using Kaplan-Meier survival curves. The Logrank test (survdiff) of package survival [53] was used to determine if survival curves were different. Other R Packages used for data analysis and visualization: ggplot2 [54], readr [55], viridis [56], and ggfortify [57]. Significance level (p value) of statistical tests is indicated by ns for non-significant, * for p<0.05, ** for p<0.01, *** for p<0.001, and **** for p<0.0001.

## Results

### Synthetic serotype switching by genomic capture and tn7 integration

**Fig 1:**
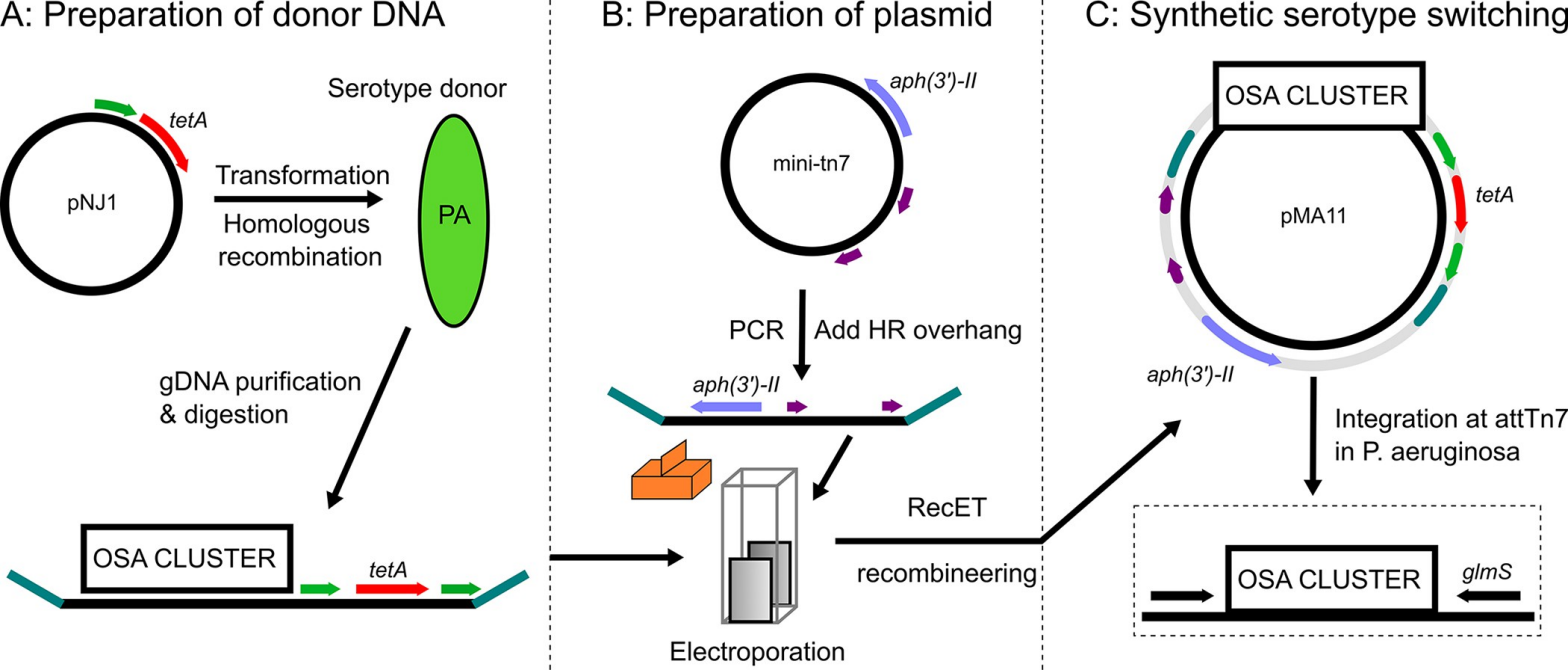
Synthetic serotype switching in P. aeruginosa by RecET genomic capture and tn7 tagging. A: Donor serotype strains are prepared by integration of pNJ1 in the serotype cluster by homologous recombination. This vector (pNJ1) contains a 900 bp PCR fragment homologous to the serotype cluster (green), tetA (red), and sacB (not pictured). Genomic DNA is purified and digested by restriction enzymes that cut up- and downstream of the serotype cluster (within 5000 bp). B: Genomic tagging plasmid (pUC18R6kT-mini-Tn7T-Km) is linearized by PCR to add 80 bp overhangs (teal) which are homologous to regions up- and downstream of serotype clusters. Serotype donor DNA and linearized plasmid are mixed and electroporated into competent Gbdir-pir cells which facilitate RecET recombineering. C: The resulting plasmid can facilitate synthetic serotype switches by integration at attTn7 in P. aeruginosa, which is upstream of glmS. DNA between Tn7L and Tn7R (purple) is integrated at attTn7 in the presence of helper plasmid pTNS2. Antibiotic genes tetA and aph(3’)-II were excised by sucrose counter selection and FLP recombination, respectively.

To investigate the impact of serotype switching in the evolution of HIRIC ST111, we designed a method that enabled the construction of isogenic strains each expressing different O-antigens (i.e. synthetic serotype switching). This method includes genetic tagging of the O-antigen (OSA) gene cluster with an antibiotic resistance gene to facilitate genomic capture, preparing a mini-tn7 vector for recombination, and RecET recombineering for the construction of a serotype switch plasmid as illustrated in Fig 1AB. The method enables the capture of the entire O-antigen cluster from a donor strain of choice, and site-specific integration at attTn7 in *P*. *aeruginosa* (Fig 1C) followed by removal of selection markers and plasmid constructs. Synthetic serotype switching is then accomplished by first deleting the native OSA cluster (16.6 kb in PA14, 22.5 kb in PAO1, and 14.3 kb in ST111) using allelic replacement (Materials and Methods) and then integrating a heterologous O-antigen cluster by tn*7* tagging. This approach was used to construct isogenic strains expressing serotypes O4 or O12 in the well-characterized strains PAO1 (serotype O5), PA14 (serotype O19) and a serotype O4 isolate of ST111, to understand the biological effect of the O4-to-O12 serotype switch in different genetic backgrounds (Table 1).

### Verification of strains expressing heterologous O-antigens

**Fig 2:**
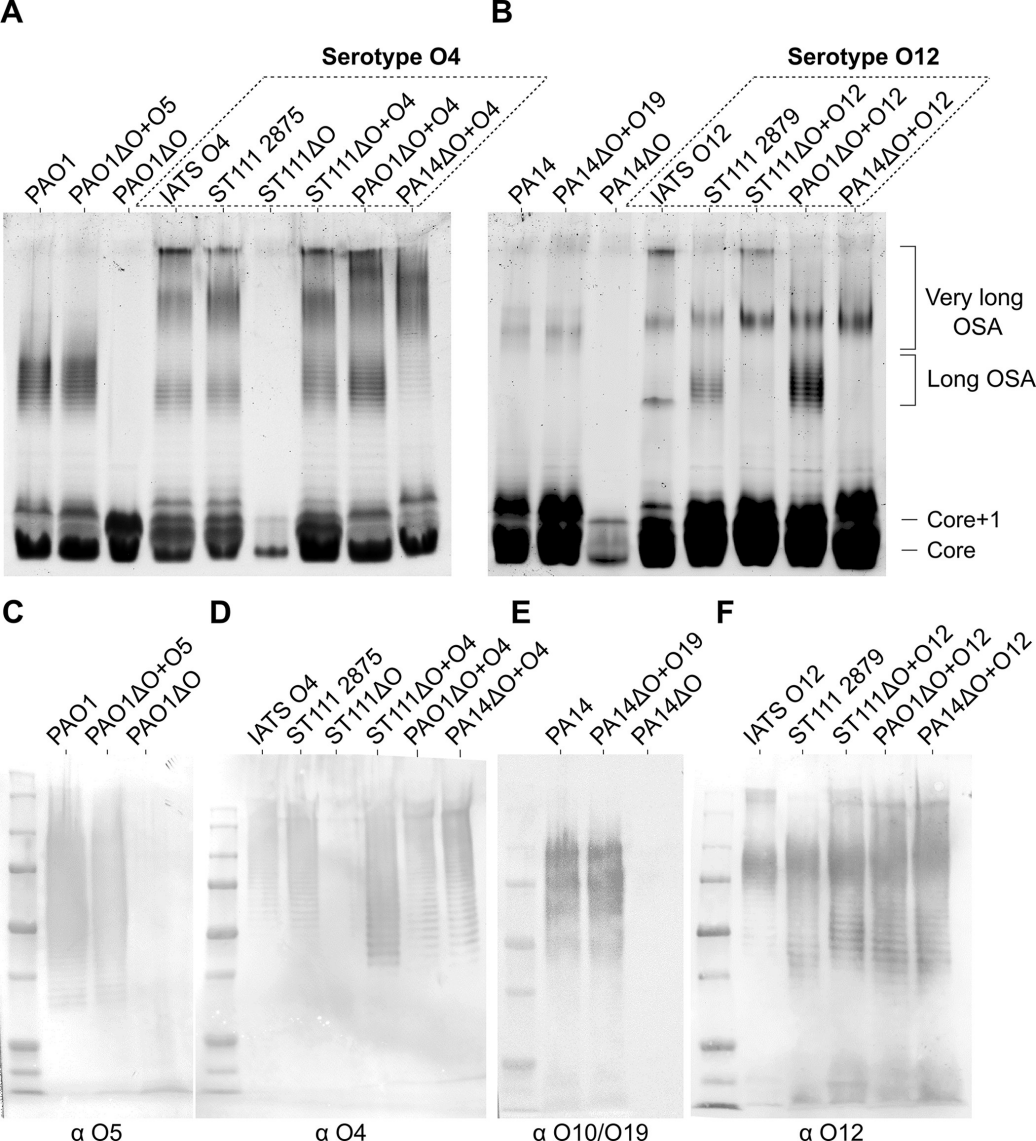
Pro-Q and Western blot LPS visualization of P. aeruginosa strains. A & B: LPS separated by electrophoresis on SDS PAGE 4–20% gels and stained with Pro-Q, strain names have been shown above their corresponding lanes. The location of different LPS glycoforms have been indicated on the gels. C-F: Western blot gels using antibodies specific for P. aeruginosa serogroups (primary antibody shown below each Western blot membrane). C: MF15-4 Mouse-IgM (monoclonal), D: Rabbit anti sero O4 (polyclonal), E: Rabbit anti sero O10/O19 (polyclonal), F: Rabbit anti sero O12 (polyclonal). For Western blots, anti-mouse IgM-HRP or anti-rabbit IgG-HRP were used as secondary antibodies, as appropriate.

To verify that our approach resulted in isogenic strains expressing heterologous O-antigens, we first purified and separated LPS, from an equal number of cells, on SDS PAGE gels to enable direct comparisons between strains expressing different O-Antigens. The LPS was visualized with the Pro-Q Emerald 300 LPS stain or by Western blotting, using antibodies specific for different O-antigen serotypes (Materials and methods). We found that all strains reacted with the LPS stain (Fig 2A and 2B) and the visualized O-antigen LPS banding indicates functional heterologous expression of OSA in all engineered strains. As expected, no LPS bands were observed in strains without an OSA gene cluster. These results were further confirmed with Western blot analyses using corresponding serotype specific antibodies (Fig 2C–2F).

This experiment also demonstrate that expression of the O-antigen gene cluster is unaffected by its location on the chromosome (i.e. the native location as found in wildtype strains versus a location at the Tn7 attachment site in the engineered strains). For example, both wildtype PA14 and strain PA14ΔO+O19 produce O19 O-antigen, which can be detected using O19-specific antibodies (Fig 2F). In the latter strain, the native O19 OSA gene cluster has been deleted, followed by insertion of the same gene cluster in the Tn7 attachment site (c.f. Fig 1). As shown in Fig 2F, the O19 antigen detected in PA14ΔO+O19 is similar to wild type PA14 in terms of the amount and chain lengths detected, demonstrating that the engineered strains produce the expected O-antigen and that expression from the Tn7 site does not interfere with the O-antigen production process.

However, some differences between the strains were observed. For example, it was evident that the OSA length regulation is dependent on the genetic background of the strains used for heterologous expression. As seen in Fig 2A and 2B, the LPS size distribution is different among most of the strains. In IATS O12 the “very long” OSA chains seems to be the predominant LPS glycoform, whereas in ST111, PA14, PAO1 there is a mix of “long” and “very long” OSA chains. This glycoform diversity between genetic backgrounds is to be expected as the gene that regulates the “very long” OSA chains resides outside the OSA locus [58].

Lastly, genome sequencing of the engineered strains confirmed the integrity of OSA gene cluster and that no genetic changes had been introduced to the heterologous OSA cluster during strain construction (S1 Table). When combined, these results show that the constructed strains are isogenic and able to produce the expected type and amounts of different O-antigens.

### Serotype O12 adheres better to tissue culture polystyrene than O4

We first investigated if expression of different O-antigens affects the ability of the strains to adhere to tissue culture polystyrene microtiter plates. To do this, we used a variant of the classical crystal violet assay [43] wherein washed cell suspensions were incubated for 2 hours in microtiter plates without nutrients. After removal of planktonic and weakly attached cells, the adhering cells are quantified by crystal violet staining.

**Fig 3:**
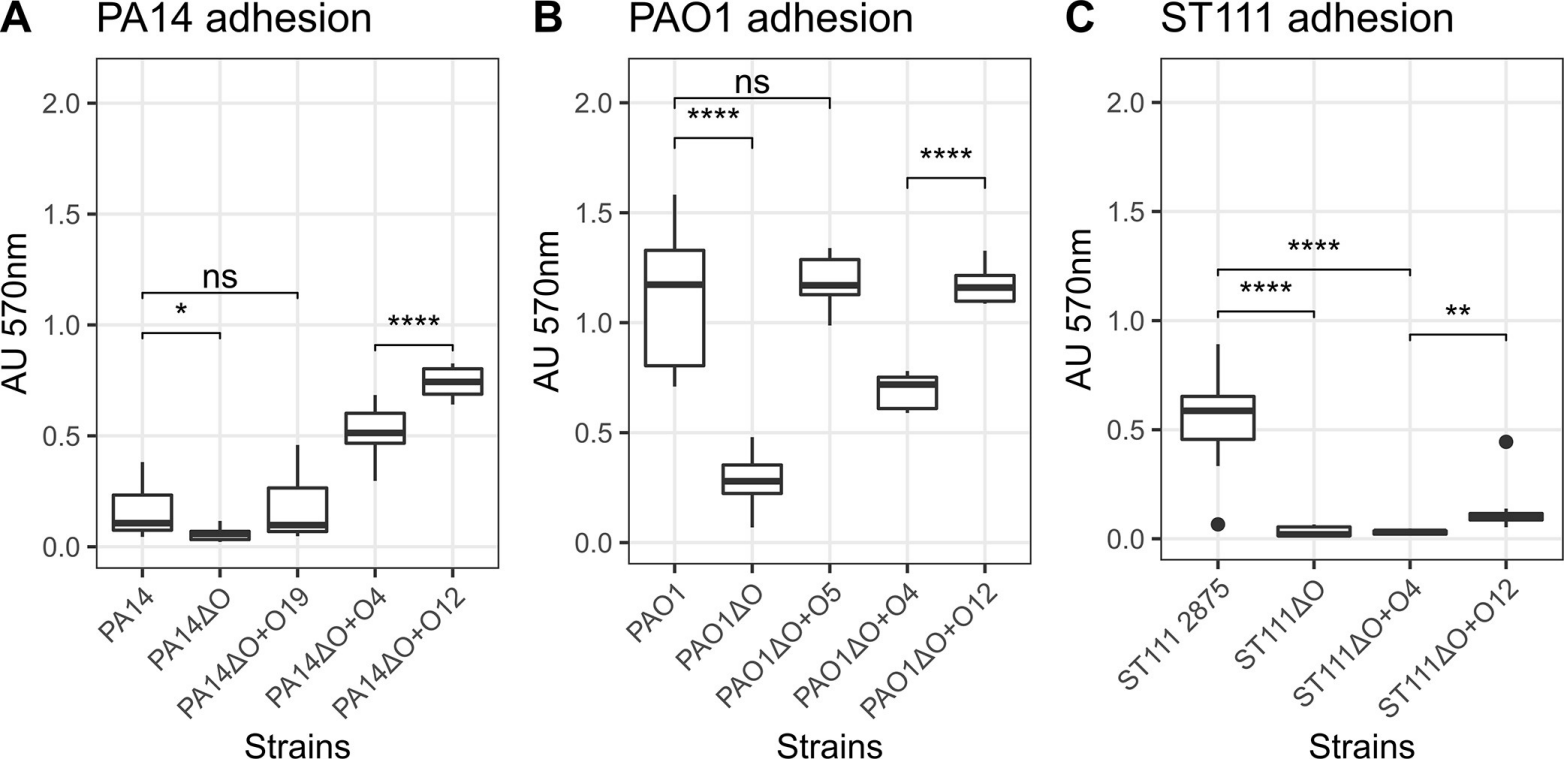
O-antigen structure changes adhesion of strains in PA14 (A), PAO1 (B), and ST111 (C). The adhesion of strains (shown on y-axis, common to all plots) was measured after 2 hours by crystal violet staining. All strains were characterized using 12 biological replicates and 6 technical replicates. Common for all strains is that loss of the O-antigen severely impairs the ability of the bacteria to adhere to tissue culture polystyrene plates (p>0.05), and complementation with the wild-type serotype restores adhesion in PA14 (O19) and PAO1 (O5). Surprisingly this is not the case for ST111 (O4) where all of the engineered strains show impaired adhesion compared to the wild type. For all strains tested we find that serotype O12 strains are significantly more adherent than serotype O4 strains (p>0.05).

Using this assay, we find that the three wild-type strains (PA14, PAO1, and ST111) exhibited different levels of adhesion (Fig 3), and that deletion of the OSA gene cluster resulted in a significant decrease in adhesion for all strains (cf. wildtype and ΔO (OSA deletion strains) strains in Fig 3). For PA14 and PAO1, we find that heterologous expression of OSA restores adhesion, but that only expression of O12 in ST111 results in an adhesion phenotype distinguishable from the OSA deletion strain. The results obtained from ST111 suggest that heterologous expression of either O12 or O4 could not complement the adhesion phenotype of the OSA deletion strain to reach wild type (O4) levels. This was not due to lack of OSA production (Fig 2), and we speculate that this phenotype is a result of mutations that inactivate other (non-OSA) adhesion factors.

Importantly, in all strain backgrounds we find that heterologous expression of O12 is associated with significantly higher adhesion than O4 expression. Specifically, in the PA14 background we find that serotype O12 is 1.4x more adherent than serotype O4 (*p* = 2.2e-5, multiple t-test w/ fdr) and that serotype O12 is 1.7x more adherent than the O4 strain (*p* = 5e-12, multiple t-test w/ fdr) in PAO1. Despite the lowered adhesion capabilities of all engineered ST111 strains, we nevertheless find that O12 is 4.3x more adherent than the engineered O4 strain (*p* = 9.2e-3, multiple t-test w/ fdr)

Based on the results of these experiments we conclude that the molecular structure of the O-antigen has a significant effect on the early stages of biofilm formation (adhesion) as demonstrated by attachment to tissue culture polystyrene, and that serotype O12 facilitates better adhesion than O4 regardless of the genomic background.

### Relative polarity of OSA structures plays a role in bacterial adhesion

**Fig 4:**
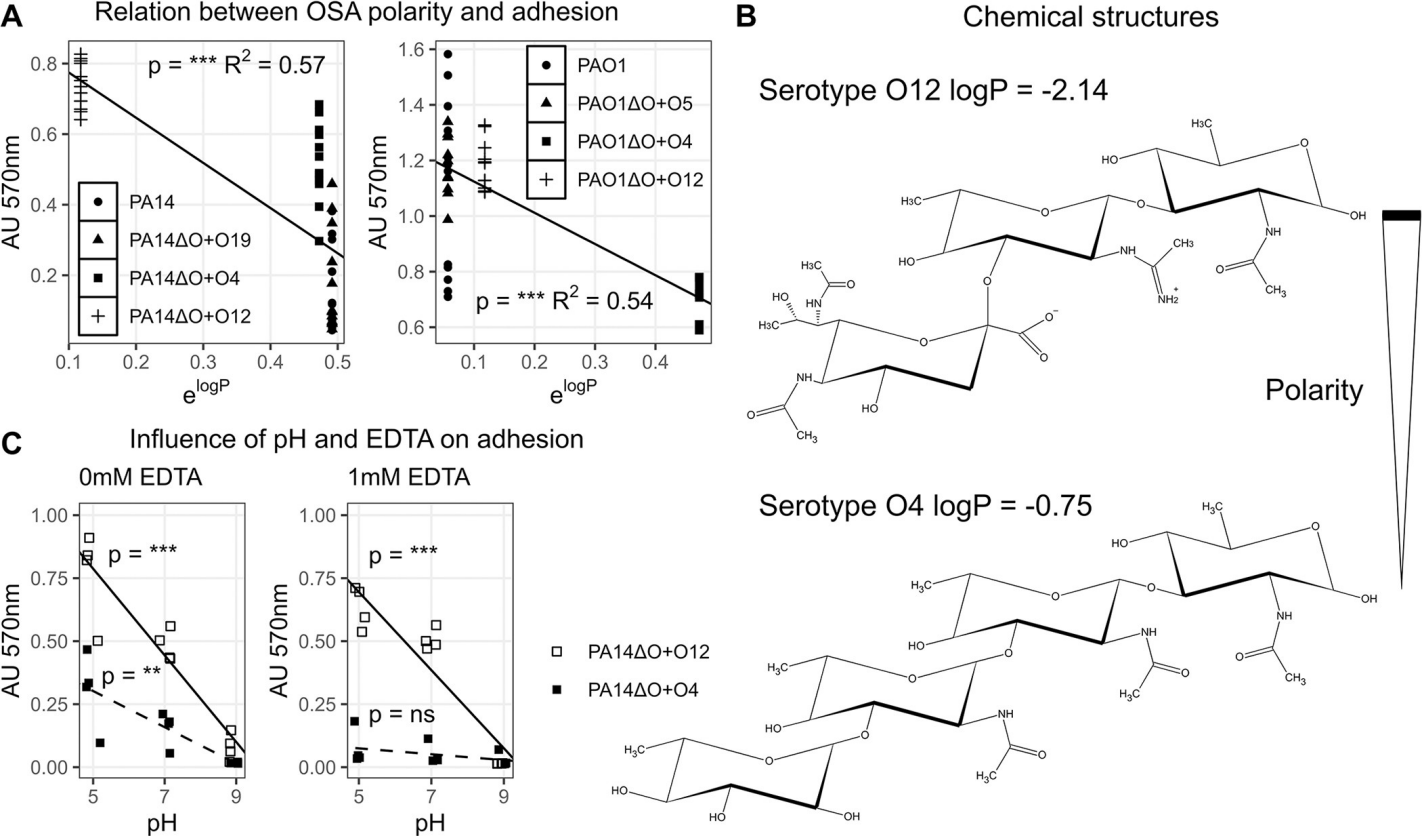
Relating the biophysical forces that govern bacterial adhesion to experimental data. A: We show that the relative polarity of the expressed serotype correlates with adhesion to tissue culture polystyrene. This is the case for PA14 (left) and PAO1 (right) strains expressing different serotypes. Significant (p and R^2^ values shown in each plot) linear models between adhesion and the polarity of OSA repeat units are made for each strain and shown as lines between the data points. B: The chemical structure of the OSA repeats for serotype O12 (top) and serotype O4 (bottom) along with their relative polarity (logP). In terms of relative polarity, these figures indicate that the O12 repeat unit is more polar than the O4 repeat unit. C: The influence of pH and EDTA on adhesion. The adhesion of PA14ΔO+O4 and PA14ΔO+O12 was assayed at pH 5, 7, and 9 in the absence (left) and presence (right) of the metal chelator 1mM EDTA. Linear models of adhesion as a function of pH were made for both strains and shown as a line, for PA14ΔO+O12, or a dashed line, for PA14ΔO+O4. Significance of linear models between adhesion and pH is shown in the figure above their corresponding lines. In the absence of EDTA, adhesion of both strains increases as pH decreases. The electrostatic repulsion between the negatively charged substrate and bacteria is reduced, as negatively charged phosphates in the LPS become increasingly protonated at decreasing pH. In the presence of EDTA, adhesion of PA14ΔO+O4 is abolished, while PA14ΔO+O12 is still able to adhere, suggesting that serotype composition plays a major role in binding LPS stabilizing divalent cations.

Bacterial adhesion is governed by many physiochemical forces including polarity [59–61]. To test the influence of polarity on the adhesion of strains to tissue culture polystyrene, we modelled the relative polarity (Partition coefficient logP = log [A]_org_-log [A]_aq_, see materials and methods) of a single OSA repeat unit for each serotype explored in this study (O4, O12 as well as parental serotypes O5 and O19 in PAO1 and PA14, respectively). In terms of relative predicted hydrophilicity, the serotypes rank O5 > O12 > O4 > O19. We find a significant association between the relative polarity of OSA structures (exponent of logP) and the experimental adhesion data of both PAO1 and PA14 (absorbance at 570nm) using linear regression models (p<0.05, t-test) (Fig 4A). While this model is not sufficient to explain all variance (R^2^ shown in Fig 4A), it shows that polarity of the OSA does play a substantial role in adhesion to tissue culture polystyrene, in a strain specific manner as PAO1 strains can be seen adhering at a higher level than PA14 strains despite expressing the same serotypes.

### Serotype O12 maintains adhesion in the presence of EDTA, whereas O4 does not

Another key aspect of bacterial adhesion is the influence of electrostatic interactions, which normally represent repulsive forces between bacteria and substrate (tissue culture polystyrene) since both are negatively charged [59,62–64]. The surface charge of *P*. *aeruginosa* is predominantly mediated by phosphate groups in the LPS core, which serve to increase surface electronegativity. When the LPS is decorated with OSA, the overall surface electronegativity is reduced by divalent cations, which stabilize the outer membrane [13,45,59,65,66]. Due to the different chemical properties of serotypes O4 and O12 (Fig 4B), we hypothesize that the adhesive properties of each serotype are differentially affected by pH and the presence of the metal chelator EDTA. To test this, we assayed adhesion of isogenic strains expressing either O12 or O4 (PA14ΔO+O4 and PA14ΔO+O12) at pH 5, 7, and 9 in the absence or presence of 1mM EDTA.

We find a significant linear correlation between pH and adhesion for both serotypes O12 and O4 (significance levels indicated in both panels of Fig 4C), showing that adhesion increases as pH decreases. We assume that the physiochemical basis for increased adhesion with decreasing pH is due a reduction in the charge of phosphate groups in the LPS [67], which in turn decreases the repulsion between the negatively charged bacteria and tissue culture polystyrene. However, in comparing the slopes for each serotype, we see that the effect of pH on adhesion is 2.4 times greater for serotype O12 (slope: -0.17) than O4 (slope: -0.07) (Fig 4C, left panel). We propose that this difference is in part due to the positively charged acetamidino group in the OSA of serotype O12 [68,69] (Fig 4B) and in part due to the ability of serotype O12 to mask its surface charge by binding more divalent cations with the carboxyl group [59] than serotype O4.

Importantly, adhesion of PA14ΔO+O4 is greatly reduced upon addition of EDTA (Fig 4C, right panel), whereas adhesion of PA14ΔO+O12 is unaffected by EDTA. We propose that the same functional groups in the OSA that differentiate these strains response to pH (acetamidino and carboxyl), can maintain adhesion in the presence of EDTA. Overall, these results show that chemical differences between O12 and O4 impact adhesion behaviors of cells as well as the sensitivity of these behaviors to changing environmental conditions.

### Cell physiology and motility behavior is modulated by the O-antigen composition

**Fig 5:**
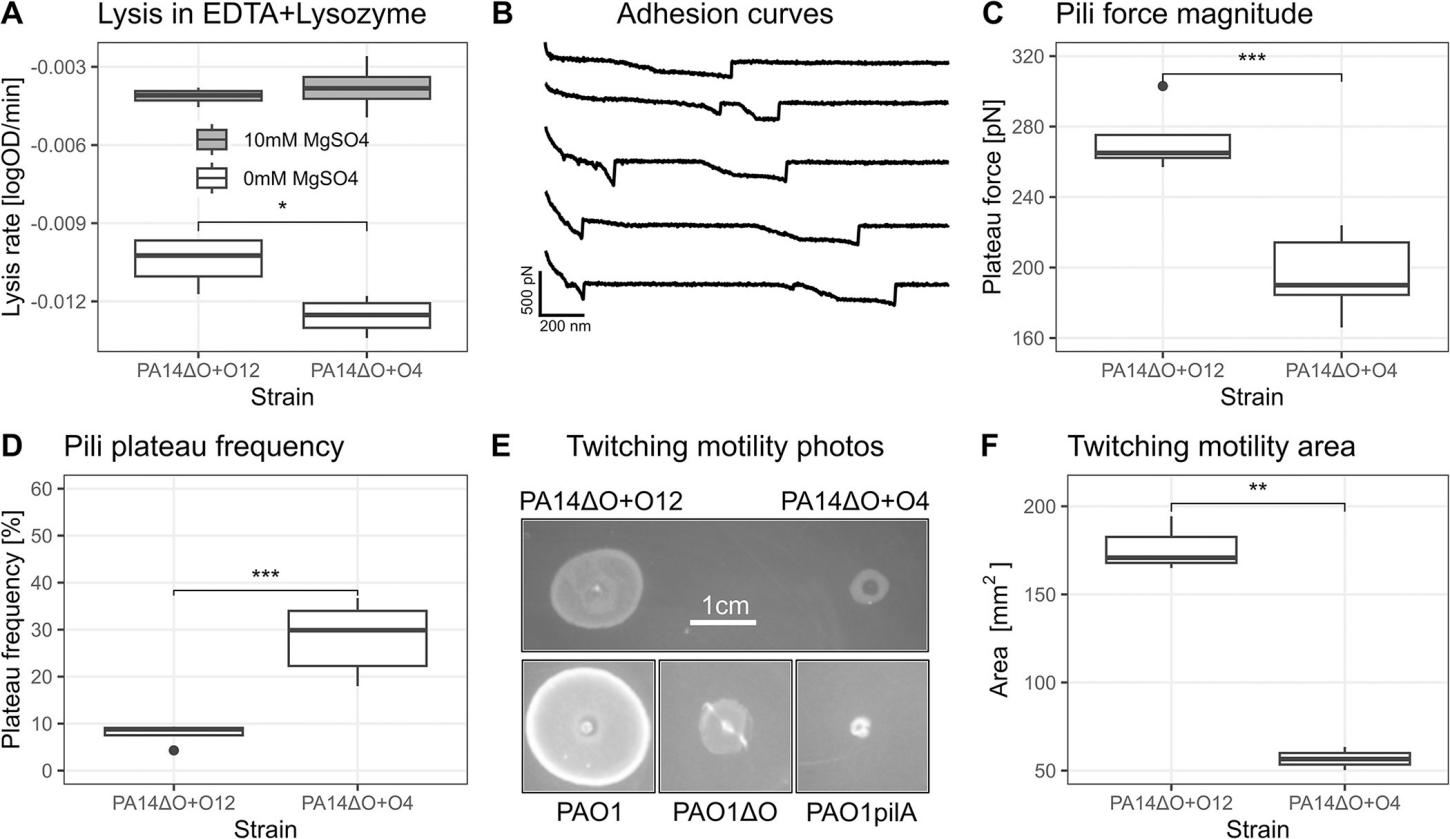
OSA composition plays a role in a multitude of bacterial interactions not directly related to LPS. A: The serotype significantly affects the rate of lysis when treated with 1 mM EDTA & lysozyme. This effect can be rescued by the addition of magnesium, implicating the role of divalent cations in this interaction. Each boxplot represents data from 4 biological replicates. B-D: Atomic force microscopic (AFM) characterization of serotype switched strains shows that pili mechanical properties are significantly affected by serotype composition; B: Representative adhesion curves for PA14ΔO+O4 from which we can identify type IV pili by their characteristic plateau signatures. C: Strength and D: frequency of force plateaus for PA14ΔO+O12 and PA14ΔO+O4 strains. E: Serotype composition affects twitching motility as can be seen comparing the motility of PA14ΔO+O12 and PA14ΔO+O4 (grown on the same plate, inoculated simultaneously). Prior understanding about twitching motility is that the O-antigen is essential for twitching motility [73], but here we can show that this is not the case with isogenic PAO1 strains deficient in either O-antigen (PAO1ΔO) or type IV pili (PAO1pilA). Scale bar (1 cm) is common to all twitching motility photos. F: The twitching motility area is significantly affected by serotype composition.

Since our results suggest that O4 and O12 O-antigens have different capacities to bind LPS stabilizing cations (such as Ca^2+^ and Mg^2+^), we hypothesized expression of different O-antigens would affect membrane stability and consequently also the functionality of membrane associated proteins. To test this hypothesis, we first investigated if EDTA exposure would result in differential increase in outer membrane permeability in cells expressing either O4 or O12 O-antigens. *P*. *aeruginosa* is known to be highly resistant to lysozyme, but becomes lysozyme-sensitive when co-treated with EDTA which makes the outer membrane of *P*. *aeruginosa* permeable [45]. Measurements of the lysis rate of cultures exposed to 1 mM EDTA and 10 mg/mL lysozyme revealed that PA14ΔO+O12 is less sensitive to the treatment than PA14ΔO+O4, suggesting that the membrane stability is influenced by the chemical composition of the O-antigen (Fig 5A). We additionally show that addition of magnesium restores membrane stability (and thus resistance to EDTA+lysozyme treatment), which confirms the role of divalent ions in this interaction.

If membrane stability is affected by the chemical composition of O-antigen, it could be expected that the function of proteins or protein complexes associated with the membrane would also be affected by different O-antigen structures. To explore this idea, we focused on Type IV pili (T4P) and used atomic force microscopy (AFM) to investigate at single-cell level, the mechanical properties of T4P in cells expressing different O-antigens.

To quantify the forces between *P*. *aeruginosa* polystyrene substrates, we used single cell force spectroscopy (SCFS) [48,70]. A single bacterial cell is attached to an AFM colloidal probe and brought into contact with a polystyrene surface, while recording force-distance curves of the approach and retraction of the AFM cantilever. The adhesive interactions observed in the retraction curves are established through interactions between biological polymers (proteins, polysaccharides) at the surface of the bacterium and the polystyrene surface. The features observed in the adhesion curves are very often specific of certain types of biological structures, such as the type IV Pili force plateau signature [47,50]. When characterizing the adhesion of PA14 strains (PA14ΔO+O4 and PA14ΔO+O12) to polystyrene, we identified the relative contribution of type IV pili through force plateaus (Fig 5B) and found a significant difference in the adhesion force of pili expressed by the PA14ΔO+O12 (284 ± 37 pN) strain compared to PA14ΔO+O4 (195 ± 39 pN) (Fig 5C). Interestingly, the frequency of force plateaus is lower for the PA14ΔO+O12 strain (8% vs 28%) (Fig 5D), which may indicate that type IV Pili expressed by the PA14ΔO+O12 strain are mechanically more stable than those expressed by the PA14ΔO+O4 strain. This result supports a role for O-antigen composition in the function of the type IV pili and potentially other membrane associated proteins through its role in stabilizing the outer membrane of the bacteria.

T4P are known for its essential role in twitching motility, which allows *P*. *aeruginosa* to move across solid or semisolid surfaces by the extension and retraction of pili [46,71,72]. As we have shown that the serotype composition alters the biophysical function of pili, we next tested if O-antigen composition affected twitching motility phenotypes. We found that the twitching motility area of PA14ΔO+O4 is substantially reduced compared to PA14ΔO+O12 (Fig 5E and 5F). We note that twitching motility is not abolished in the O4 or OSA deficient strains as observed in a twitching motility deficient PAO1pilA mutant (Fig 5E) (additional twitching motility data shown in S1 Fig). Overall, these results confirm that the serotype composition can directly or indirectly alter cell physiology and bacterial behaviors that depend on the function of membrane-associated protein complexes.

### Serotype composition plays a major role in a murine acute pneumonia infection model

**Fig 6:**
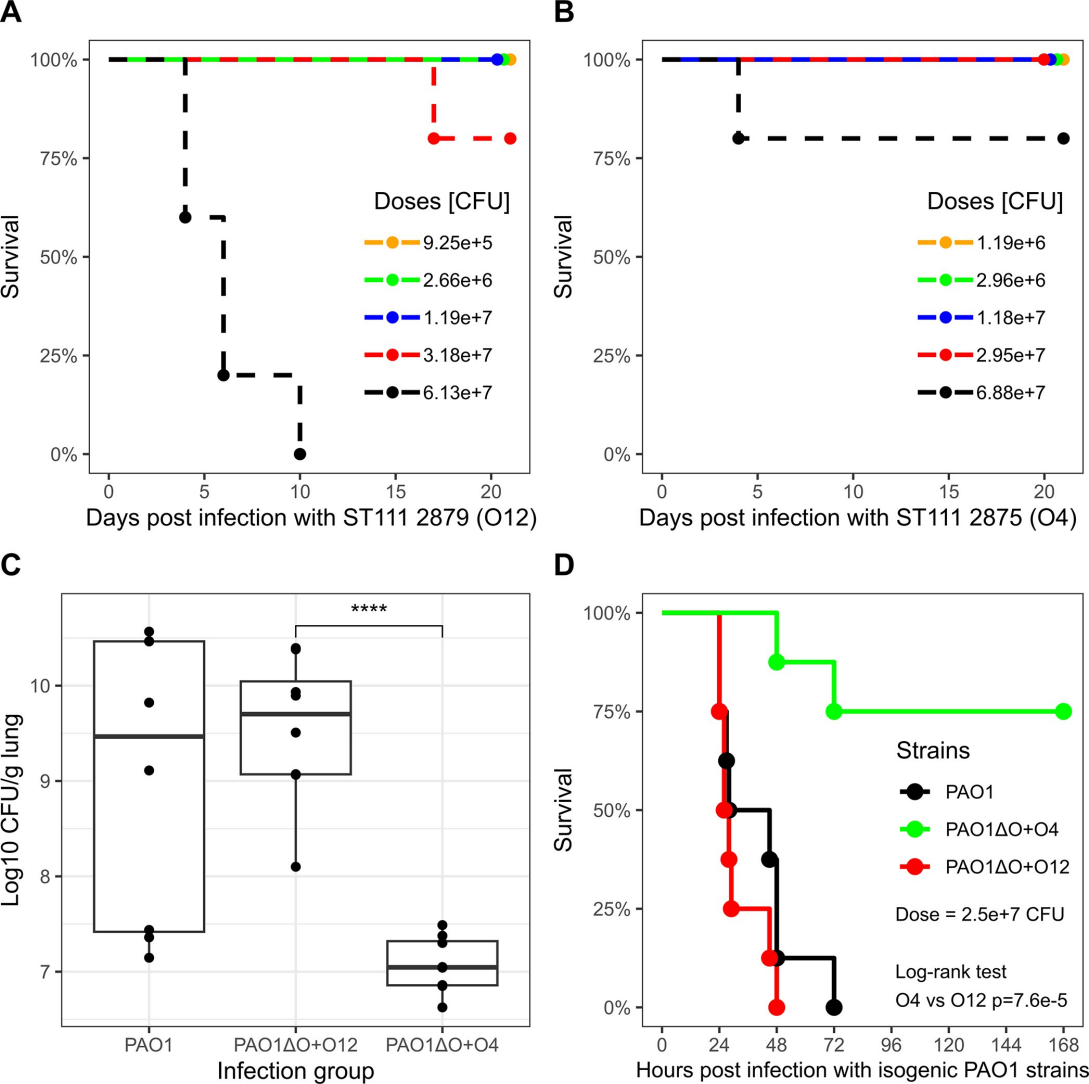
Virulence characterization in clonal and isogenic serotype strains of P. aeruginosa expressing different O-antigens. Groups of mice were infected intranasally with the indicated dose of bacteria in an acute pneumonia infection model using BALB/c mice. A & B: Multiple groups of mice (n = 5) infected with a clinical strain ST111 2879 expressing serotype O12 (A) or ST111 2875 expressing serotype O4 (B) monitored for survival 20 days post infection. C: 24h post-infection (dose ~ 2.5e+7 CFU), whole lungs were aseptically collected from euthanized animals (n = 8) and homogenized. CFU was estimated from homogenates by plate counting on selective media (PIA). D: Mice infected (n = 8) with isogenic PAO1 strains expressing different O-antigens were monitored for survival 7 days post infection.

Given the differential impact of O-antigen structures on adhesion phenotypes and membrane stability (which influences the sensitivity towards lysozyme and T4P-dependent motility phenotypes), we next investigated the impact of O-antigen structures on virulence. We first assessed the virulence of clinical ST111 strains expressing different serotypes in a murine acute pneumonia infection model using BALB/c mice. Multiple groups of mice were intranasally infected with a range of different infection doses using ST111 2875 (expressing O4) and ST111 2879 (expressing O12) (Materials and methods). Unlike PAO1 infections, where mice develop acute pneumonia symptoms by 24–48 hours post infection (see below), we observed that ST111 strains exhibit low virulence in this model. Specifically, we found that only mice infected with a high dose (approx. 6e7 CFU) of ST111 serotype O12 strain displayed delayed onset of symptoms and succumbed to infection starting day 5 post infection (Fig 6A). At the same high infection dose, ST111 2875 (expressing O4) was less virulent (Fig 6B). Although these results indicate that O12 expressing ST111 strains are more virulent that O4 strains, we decided to use the virulent PAO1 strain instead of ST111 in further experiments to corroborate this observation. To this end, we assessed the virulence of isogenic serotype-switched PAO1 strains in the murine acute pneumonia infection model. Unlike ST111, PAO1 is virulent and well studied in this model, and the use of isogenic PAO1 strains in contrast to clinical ST111 strains also exclude the possibility of unknown genetic differences which potentially may have contributed to the dissimilar virulence phenotypes observed between ST111 strains 2875 and 2879. Using PAO1 strains, mice were intranasally infected with approximately 2.5e+7 CFU and either euthanized 24 hours post-infection to estimate lung colonization (Fig 6C), or monitored for survival up to 7 days post-infection (Fig 6D).

To determine whether the serotype composition impact the infection and colonization outcome, we monitored the lung colonization 24 hours post-infection. Higher bacterial loads were recovered from mice infected with wild-type PAO1 and PAO1ΔO+O12 strains. In contrast, the bacterial burden was significantly lower in the lungs of mice infected with PAO1ΔO+O4 (Fig 6C). In comparing the virulence of PAO1, PAO1ΔO+O12, and PAO1ΔO+O4, we find that expression of serotype O4 is associated with less virulence compared to wild-type PAO1 and the O12 expressing strain. As shown in Fig 6D, mice infected with either wild-type PAO1 or PAO1ΔO+O12 succumbed to infection by 48–72 hours. In contrast, better survival (75%) was observed in mice infected with PAO1ΔO+O4 (Fig 6D). Both of these results indicate a substantial difference in virulence between expressing serotype O4 and O12.

### Composition of the OSA may play a role in serum resistance

One model to explain why serotype O12 may be more virulent than serotype O4 in the acute pneumonia infection model is that these strains exhibit an increased survival within the host. To address this, we sought to characterize the inhibition by complement-mediated killing of isogenic strains expressing different serotypes when incubated with normal human serum (NHS). Previous literature has described the importance of O-antigen length and presence in this interaction [58,74]; however, it is unknown what role the specific O-antigen composition plays.

Strains were incubated in either NHS or heat-inactivated serum (HI), as a control, for 1 hour. After incubation, the survival of each strain was quantified by CFU enumeration. We found that there is an association between loss of OSA and reduced fitness in both PAO1 and ST111 (c.f. WT and ΔO in Table 3). Complementing the PAO1ΔO strain with any serotype (O4, O12, or O5) restored survival in serum to similar levels as the wild-type PAO1 (Table 3), indicating that the chemical composition of O-antigen does not differentially affect serum resistance.

At 20% NHS, there are only subtle differences between ST111 strains (cf. ST111 2875 and ST111 2879 in Table 3). However, at 40% NHS, these differences become more pronounced and the serotype O12 strain ST111 2879 is associated with better survival in serum than serotype O4 strain ST111 2875, which may directly correlate to the observed virulence difference between these strains. Similarly, ST111ΔO+O12 is associated with 2–4 logs increased survival compared to survival of ST111 2875 or ST111ΔO+O4, suggesting that O-antigen composition plays a role in serum resistance in this genetic background.

**Table 3:** Survival of P. aeruginosa strains when incubated with normal human serum. Survival is quantified by determining the log10 fold difference between CFU recovered after 1h incubation in normal human serum and incubation in heat-inactivated serum (for 20% NHS n = 4 and for 40% NHS n = 3). NA: non-applicable; strains that do not express an O-antigen.

Strain	Serotype	Log10 fold change in CFU after exposure to 20% serum for 1 hour, 37°C	Log10 fold change in CFU after exposure to 40% serum for 1 hour, 37°C
PAO1 WT	O5	0.05	-0.04
PAO1ΔO	NA	-1.91	-2.73
PAO1ΔO+O5	O5	-0.26	-0.04
PAO1ΔO+O4	O4	-0.03	-1.22
PAO1ΔO+O12	O12	-0.29	-0.52
ST111 2879	O12	-0.03	-1.22
ST111 2875	O4	-1.02	-4.69
ST111ΔO	NA	No CFU recovered	No CFU recovered
ST111ΔO+O4	O4	-0.1	-4
ST111ΔO+O12	O12	-0.08	-1.35

## Discussion

A limited number of MDR/XDR *P*. *aeruginosa* sequence types (e.g. ST235, ST111, ST244, and ST253) are highly prevalent in hospitals worldwide. To limit the continuing spread of these clones and to predict the next waves of additional MDR/XDR *P*. *aeruginosa* high-risk clones, it is important to determine the mechanisms that enables these particular clones to succeed while others do not. In this study, we examined how different O-antigen serotypes modify *P*. *aeruginosa* pathophysiology, since serotype switching from O4 to O12 O-antigens has previously been shown to be a characteristic of the emergence and success of ST111 as a high-risk clone.

To pinpoint how expression of different serotypes affects *P*. *aeruginosa* biology, we engineered and analyzed isogenic strains each expressing either O4 or O12. We find that the sugar composition of O-antigens affect adhesion in both laboratory strains (PAO1 and PA14) and in clinical ST111 strains (Fig 3). Our results in relation to adhesion extend previous studies that have clearly demonstrated that the LPS and the O-antigen plays an important role in adhesion and the initial stages of biofilm formation by studying knockout mutants to the O-antigen and the core [59,74–78]. These studies have shown how the different LPS glycoforms of *P*. *aeruginosa* (OSA-capped, CPA-capped, uncapped) can mediate different interactions with biotic and abiotic surfaces. Here, we find that our O-antigen deficient mutant strains are the most impaired in adhesion to the hydrophilic [79] tissue culture polystyrene plates, and we expect these strains are only able to express the hydrophobic uncapped LPS glycoform, due to a lack of WbpL [17,80–84], or a truncated capped core in the case of PA14 [85]. This is in agreement with previous literature that find O-antigen deficient strains to be more hydrophobic [59,74,86]. When an O-antigen gene cluster is introduced and expressed in these mutants, we find that the relative adhesion of the engineered strains to the polar tissue culture polystyrene substrate follows the ranking O12 > O4 > ΔOSA, regardless of strain genotype. We propose that this difference in adhesion is mediated, in part, by the polarity of the O-antigen repeat unit. In support of this idea, we find that there is a significant correlation between the predicted partition coefficients (relative measure of polarity, logP) of serotype repeat units and the results from adhesion experiments. Despite the limitations of this model (i.e. it only takes the contribution of a single OSA repeat unit into account, and it does not account for charged groups and many other important physiochemical forces that we know play a role in cell adhesion [60,87]), the results suggest that *P*. *aeruginosa* adhesion properties may be predicted on the basis of the sugar composition of the O-antigen.

Importantly, we observed a serotype specific impairment of adhesion for serotype O4 when adding the metal chelator EDTA. EDTA is known to destabilize the outer membrane of *P*. *aeruginosa* by sequestering cationic metal ions from the cell envelope [13,65] and we propose that the observed difference in EDTA susceptibility (on adhesion) is the result of distinct chemical properties of each OSA structure (i.e. O4 versus O12). We further substantiate this finding by showing that the rate of EDTA-mediated lysozyme lysis of these strains is different and that serotype O4 is associated with a significantly higher rate of lysozyme lysis than serotype O12. The chemical differences in OSA composition that we propose are responsible for this difference include the number of ionizable groups [20,68,88], polarity (as discussed in the previous paragraph), and capacity to bind LPS stabilizing cations (such as Ca^2+^ and Mg^2+^), which can have an effect on membrane stability [14,17,89].

The finding that outer membrane stability is affected by the chemical composition of O-antigen suggests that expression of O4 and O12 O-antigen would differentially impact the function of proteins or protein complexes that are sensitive to outer membrane structure and function. Indeed, using single-cell atomic force microscopy (AFM), we show that the strength (Fig 5C) and frequency (Fig 5D) of Type IV pili (T4P) is different in O4 expressing cells compared to O12. These differences in biophysical properties of T4P also manifested in reduced twitching motility of O4-expressing cells (Fig 5E and 5F). A link between O-antigen biosynthesis and twitching motility in *P*. *aeruginosa* has previously been established using knockout mutants. For example, deletion of the O-antigen ligase, *waaL*, responsible for ligating O-antigen molecules to the lipid A core, results in reduced twitching motility [73]. How O-antigen deficiency affects twitching motility is not understood. Here, we extend these results and show that expression of different O-antigen serotypes leads to differences in twitching motility. We propose that differences in outer membrane stability as a function of different sugar compositions of the O-antigen repeat structure affect the function of T4P (Fig 5A).

Finally, we characterized the virulence of serotype switched strains (both isogenic and clinical strains). We find that (compared to wild type PAO1), serotype O4 is associated with reduced lung colonization, and increased mouse survival during a murine acute pneumonia infection. On the other hand, serotype O12 is associated with more virulence and a higher lung colonization that is similar to wild type PAO1. We similarly characterized the survival of mice infected with clinical ST111 strains expressing serotype O4 or O12, which shows that the serotype O12 expressing strain is more virulent at similar doses of infection. We note that the clinical ST111 strains (ST111 2875 and ST111 2879) are not isogenic, and we cannot rule out contributions from genetic variations outside the O-antigen structure on the observed virulence phenotypes. Nevertheless, the link between serotype O12 and increased virulence in isogenic PAO1 strains, compared to serotype O4, suggests that the serotype configuration also contributes to the virulence of ST111.

A possible explanation for this difference in virulence between serotypes is that they confer different levels of resistance to host defenses such as complement-mediated killing. We tested the serum susceptibility of the engineered *P*. *aeruginosa* strains and found that serotype O12 is associated with increased survival in serum, but only in the ST111 genetic background. However, we also see that the engineered ST111 strains show a higher fitness in serum compared to the wild type strain ST111 2875. As the OSA of serotype O12 contains a terminal sialic acid residue, we speculate that this could modulate host immune response or mediate specific interactions that enhance virulence [90–93]. However, this requires future analysis of the host immune response to an infection with serotype switched strains.

Overall, our study reveals that *P*. *aeruginosa* pathogenicity traits such as adhesion, twitching motility, virulence, and tolerance to host defence molecules (i.e lysozyme) and complement-mediated killing are affected by the different sugar composition found in O4 and O12 O-antigens. We tracked these effects to differences in the biophysical properties of O4 versus O12 O-antigens, and show that expression of O4 affects the stability of the outer membrane, EDTA sensitivity, and strength of the Type IV pili. Importantly, these results provide clarifications on the potential selective advantages of the serotype switch from O4 to O12 that underlie the emergence and global dissemination of ST111 O12. We note that a clinical relevant aspect of ST111 O12 is the clear association between this clone and MDR/XDR profiles [5–8]. To address the possibility that changes in O-antigen expression could alter the antibiotic susceptibility of the strain (and thus contribute to the selective advantages that underlie the global success of ST111 O12), we determined the minimum inhibitory concentration (MIC) of clinically relevant antibiotics (ciprofloxacin, meropenem, tobramycin and colistin) against strains engineered to express either O4 or O12 (S4 Table). Regardless of the serotype expressed in PA14, similar MIC values were observed for these antibiotics. The same absence of a relationship between MIC and serotype expression was observed for the clinical ST111 strain, which in contrast to PA14 was resistant towards ciprofloxacin, meropenem, and tobramycin (S4 Table). As no change in susceptibility was observed when switching from O4 to O12 expression, these results suggest that antibiotic resistance is not directly linked to the serotype, and that switching from O4 to O12 expression does not contribute to the MDR/XDR profiles of ST111 O12.

Interestingly, the serotype switch from O4 to O12 appear to be specifically associated to ST111 although serotype switches to O12 from other serotypes have been observed at low frequency in other clone types, such as ST253 and ST244 [9]. It is possible that unknown interactions between host genotype and different OSA biosynthesis gene clusters restrict dissemination of the O12 OSA gene cluster across a broader range of clone types. Our observation that the OSA length regulation is dependent on the genetic background of strains used for heterologous expression (Fig 2), suggests that such interactions indeed exist. Thus, it is likely that interactions between host-specific genetic factors and OSA may affect fitness in particular environments and thereby contribute to restrict serotype switching. In support of this conclusion, recent studies have found that different, natural *Pseudomonas* isolates with identical O-antigen biosynthesis gene clusters exhibit varying sensitivities towards tailocins [94]. Future investigation of interactions between host genotype and O12 OSA expression may benefit from the recombineering method described in the present work to capture, integrate and express the O12 gene cluster across different *P*. *aeruginosa* hosts. In this context, it would be of specific interest to investigate the effects of O12 expression in the widespread XDR ST175 clone. This clone natively express the O4 serotype and is associated with reduced virulence in the *Caenorhabditis elegans* infection model [95,96]. Engineering serotype switched ST175 clones (from O4 to O12) will enable prediction of the virulence effects of a potential future serotype switch in this epidemic and highly resistant clone type.

More broadly, the genetic approach described here may be used to explore how the 20 different O-antigen structures found in *P*. *aeruginosa* impacts its physiology and behaviors. Importantly, such systematic and comparative analyses of the different O-antigens may help to better understand why certain serotypes (such as O6, O1, and O11) are prevalent in clinical settings while others are not. Such comparative studies are also needed to reveal why O12 –while required for the success of ST111—is not one of the most predominant serotypes worldwide [24,97].

Overall, our demonstration that differences between the O4 and O12 O-antigen structures can result in substantial differences in *P*. *aeruginosa* phenotypes suggest that a systematic analysis of O-antigen structure/function relationship may provide new insight into the biology of *P*. *aeruginosa*, and contribute to refine development of anti-pseudomonas antimicrobials that targets or involves LPS O-antigen molecules.

## Supporting information

S1 Fig Twitching motility of *P*. *aeruginosa* strains after incubation at 37°C for 24h in a humidified chamber. Twitching motility of n = 3 (and n = 6 for PAO1ΔO+O12 and PAO1ΔO+O4) cultures was measured and used to calculate twitching motility area for each strain. We found that expression of serotype O12 was associated with wild-type levels of twitching motility, whereas PA14ΔO+O4 exhibited significantly reduced twitching motility. We can also show that OSA deficiency (PA14ΔO and PAO1ΔO) is associated with a significant reduction in twitching motility. Finally, we show that twitching motility was abolished in a PilA deficient mutant (PAO1pilA).
(TIFF)

S2 Fig Lysis of PA14 strains in EDTA+Lysozyme in the absence of magnesium at EDTA concentrations from 0-1mM EDTA with a constant concentration of lysozyme (materials and methods). Each boxplot represents data from 4 biological replicates measured over 1 hour at 30°C. This shows that the lysis rate of P. aeruginosa in this system increases with the concentration of EDTA. In this experiment, the OSA plays a major role in resistance towards EDTA+Lysozyme mediated lysis, as a OSA deficient mutant (PA14ΔO) is very susceptible to this treatment, while OSA expressing strains are less susceptible. Moreover, we can detect subtle differences in lysis rate depending on the OSA composition and find significant differences between O4 and O12 expressing strains. This shows that the serotype composition plays a role in binding membrane stabilizing cations, which enables *P*. *aeruginosa* to resist EDTA+lysozyme lysis.
(TIFF)

S3 Fig Lysis of PA14 strains in EDTA+Lysozyme supplemented with 10mM MgSO4 at EDTA concentrations from 0-1mM with a constant concentration of lysozyme (materials and methods). Each boxplot represents data from 4 biological replicates measured over 1 hour at 30°C. Addition of MgSO4 to the EDTA+Lysozyme system abolishes all lysis in this system, which confirms that *P*. *aeruginosa* resists EDTA mediated lysis by stabilizing its outer membrane with divalent cations.
(TIFF)

S4 Fig AFM characterization of strains adhesion to glass. A) Strength and B) frequency of force plateaus for strains PA14ΔO+O12 and PA14ΔO+O4. We found no significant difference between these strains’ adhesion to glass (p > 0.05).
(TIFF)

S5 Fig Virulence characterization of *P*. *aeruginosa* strains, serotype switched (PAO1ΔO+O12, PAO1ΔO+O4) and their serotype cluster donors (IATS O12, IATS O4). Groups of BALB/c mice (n = 8) were infected intranasally with the indicated strain at a dose of 2.5e+7 CFU. A) 24 hours post-infection, lungs were aseptically collected from euthanized animals and P. aeruginosa CFU was determined by plate counting on selective media (PIA). B) The mice were monitored after infection for survival 7 days after infection.
(TIFF)

S6 Fig The adhesion of strains PA14ΔO+O12 (left) and PA14ΔO+O12-2 (right) to tissue culture polystyrene (n = 4) cannot be distinguished (p = 0.47, t-test). The strain PA14ΔO+O12 has a large deletion (26.7kb) in the region PA14R29, whereas this deletion did not occur in PA14ΔO+O12-2.
(TIFF)

S7 Fig Construction of deletion construct to facilitate deletion of entire OSA cluster in *P*. *aeruginosa*. The location of annotated genes is shown as filled blue arrows, primers as filled black arrows, and restriction sites below the horizontal line. The region shown in the top panel represents 25 kb in the PA14 genome and the relative location and size of annotated genes is to scale. DNA (horizontal black line) from a single boiled colony is used as a template for two separate PCR reactions to amplify regions up- and downstream of the OSA cluster. The resulting PCR products are purified and used as template for SOE PCR (2nd PCR round), which produces a 5.6 kb PCR product. The final construct is digested using SacI and XbaI, which enables ligation to allelic replacement vector pNJ1.
(TIFF)

S1 Table Comparison of cloned OSA clusters to source genome by BLAST alignment. Only genes and intergenic regions adjacent to the OSA cluster are compared to their ancestral genome.
(DOCX)

S2 Table Comparative genomics between serotype switched engineered strains, analyzed by breseq. (DOCX)

S3 Table List of primers. (DOCX)

S4 Table MIC of different antibiotics for *P*. *aeruginosa* ST111 and PA14 strains expressing different serotypes. The values were generated from E-test. E. coli ATCC25922 and E. coli NTCT13846 were used for control to COL MIC. E. coli ATCC25922 MIC = 0,250 μg/mL and E. coli NTCT13846 MIC = 4,000 μg/mL.
(DOCX)

S1 Supplementary Methods Integrity of cloned OSA clusters and maintenance of genotype after cloning. (DOCX)

S1 Datasheet Comparative genomics. (XLSX)
