# Supplementary figures and images for: Serotype switching in Pseudomonas aeruginosa ST111 enhances adhesion and virulence
Source: PLoS Pathog. 2024 Dec 2;20(12):e1012221. doi: 10.1371/journal.ppat.1012221 (PMC11637443; doi:10.1371/journal.ppat.1012221)

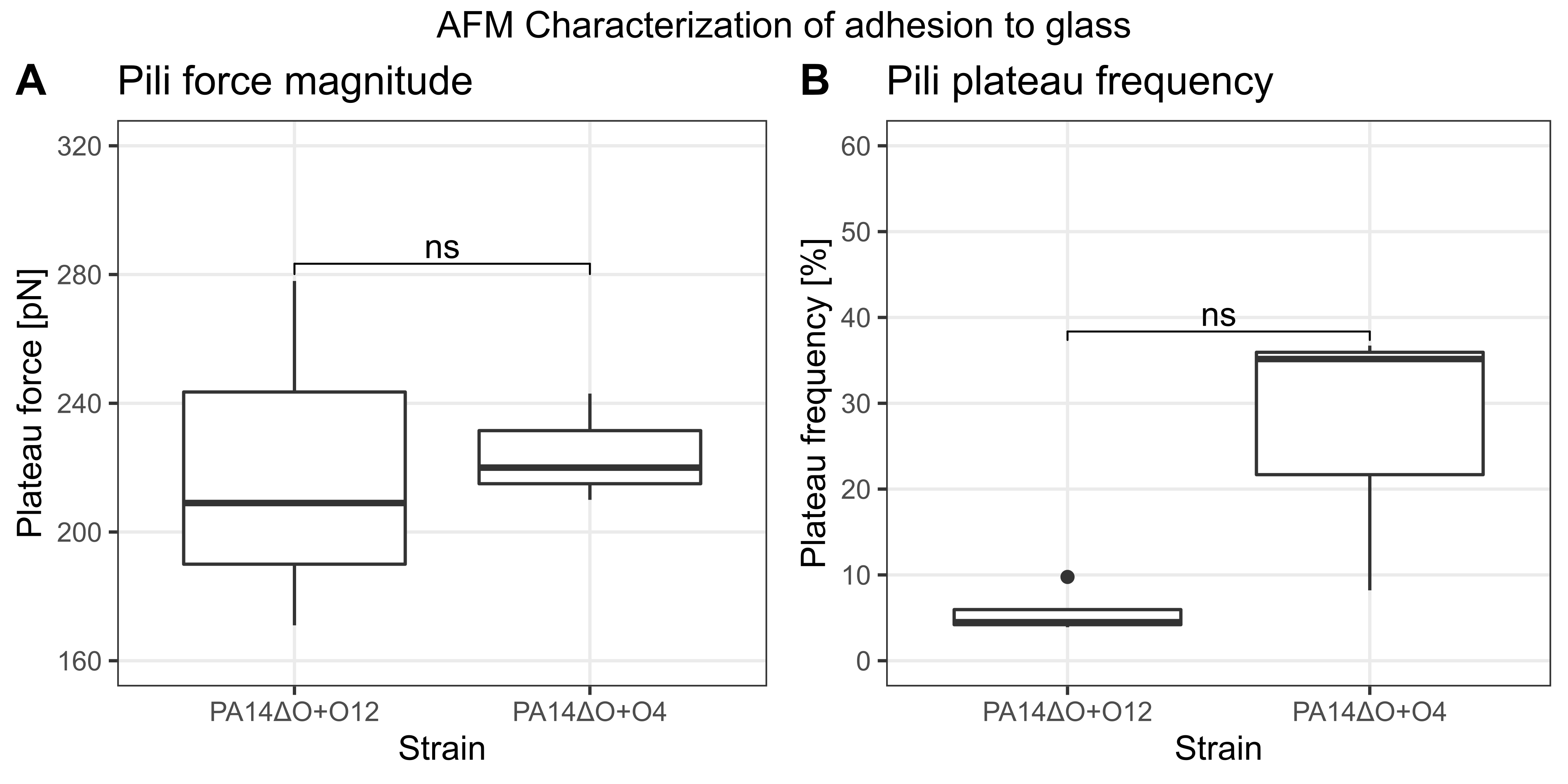

Supplement: S4 Fig — A) Strength and B) frequency of force plateaus for strains PA14ΔO+O12 and PA14ΔO+O4. We found no significant difference between these strains’ adhesion to glass (p > 0.05). (TIFF) [file ppat.1012221.s004.tiff]

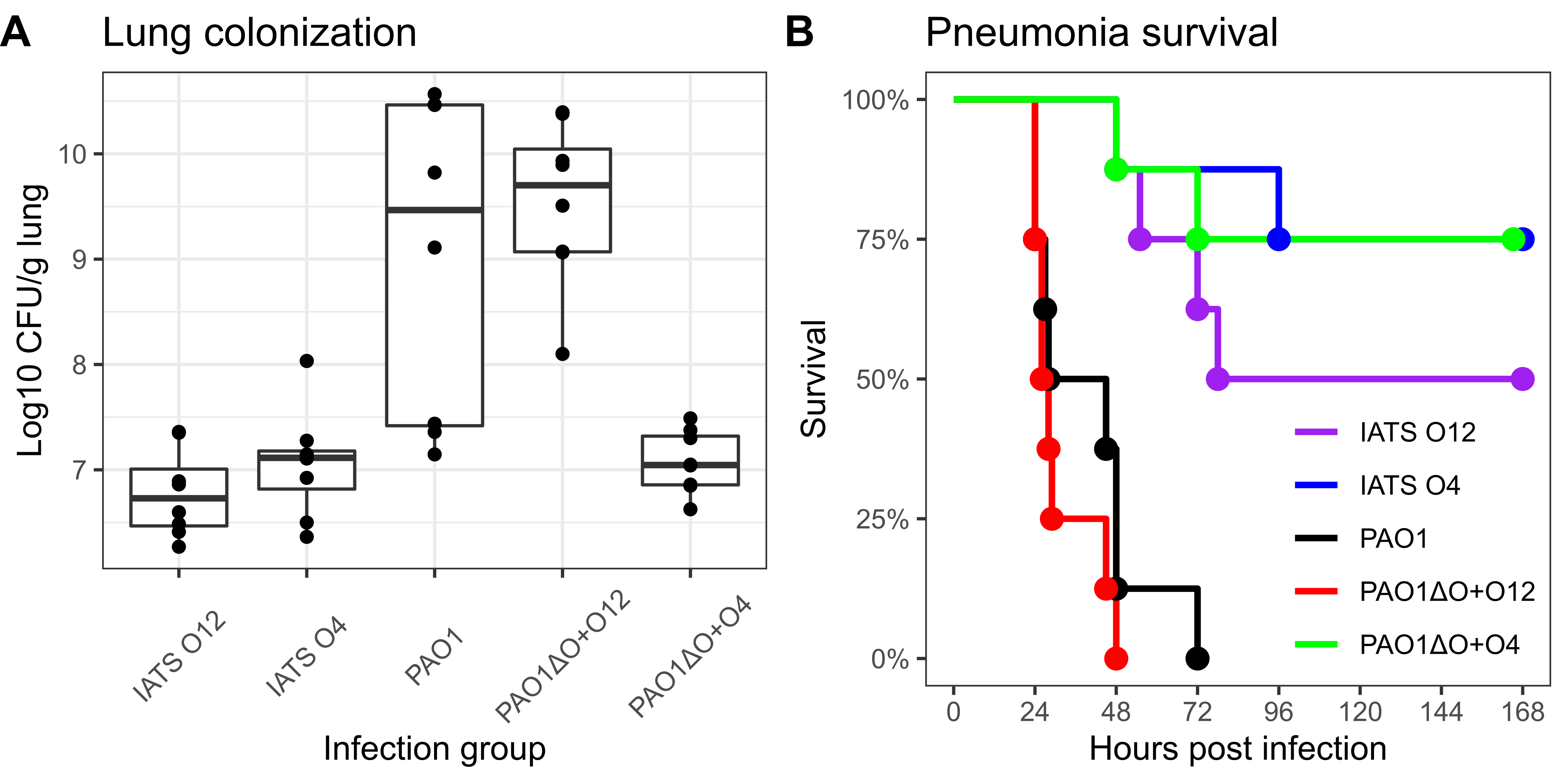

Supplement: S5 Fig — Groups of BALB/c mice (n = 8) were infected intranasally with the indicated strain at a dose of 2.5e+7 CFU. A) 24 hours post-infection, lungs were aseptically collected from euthanized animals and P. aeruginosa CFU was determined by plate counting on selective media (PIA). B) The mice were monitored after infection for survival 7 days after infection. (TIFF) [file ppat.1012221.s005.tiff]

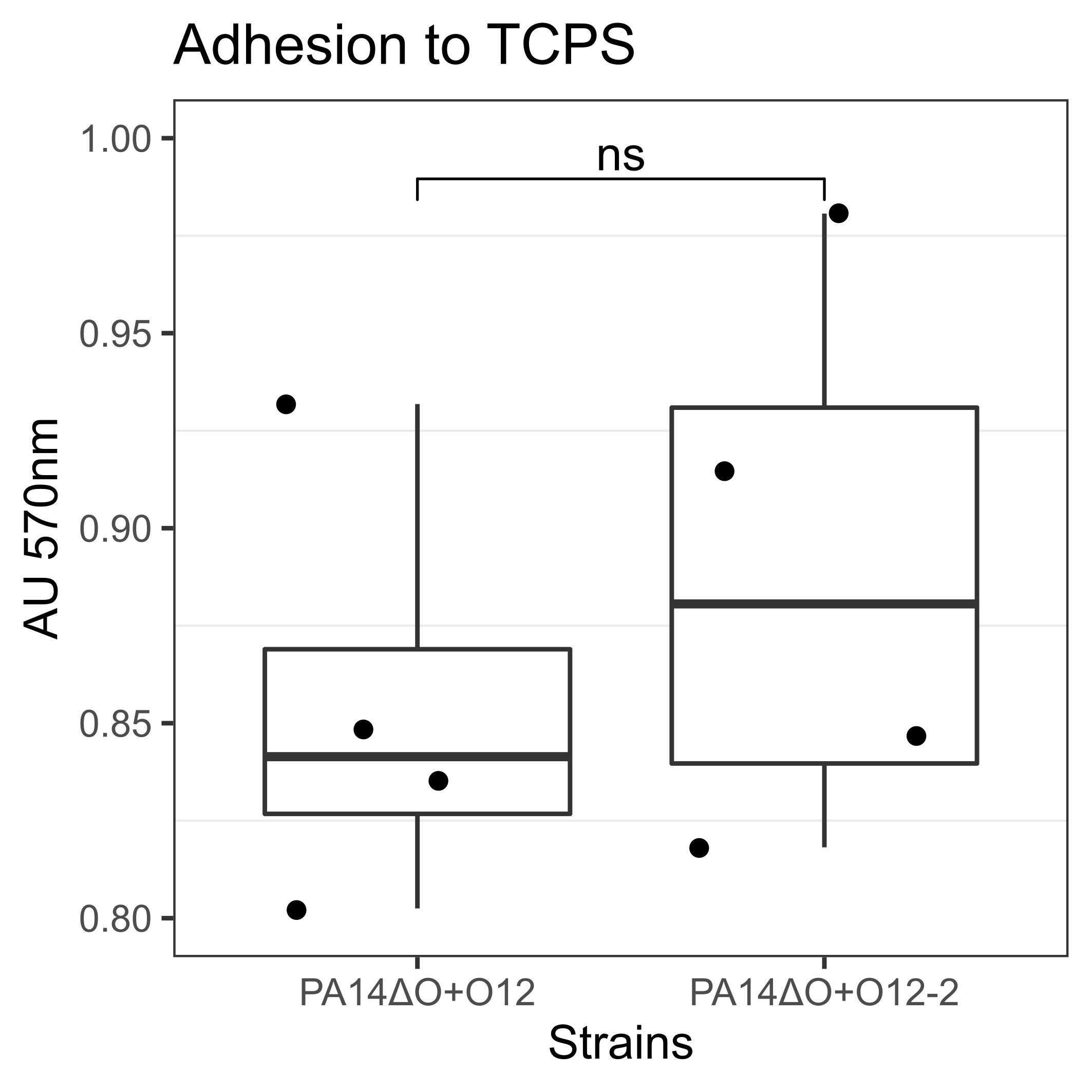

Supplement: S6 Fig — The strain PA14ΔO+O12 has a large deletion (26.7kb) in the region PA14R29, whereas this deletion did not occur in PA14ΔO+O12-2. (TIFF) [file ppat.1012221.s006.tiff]

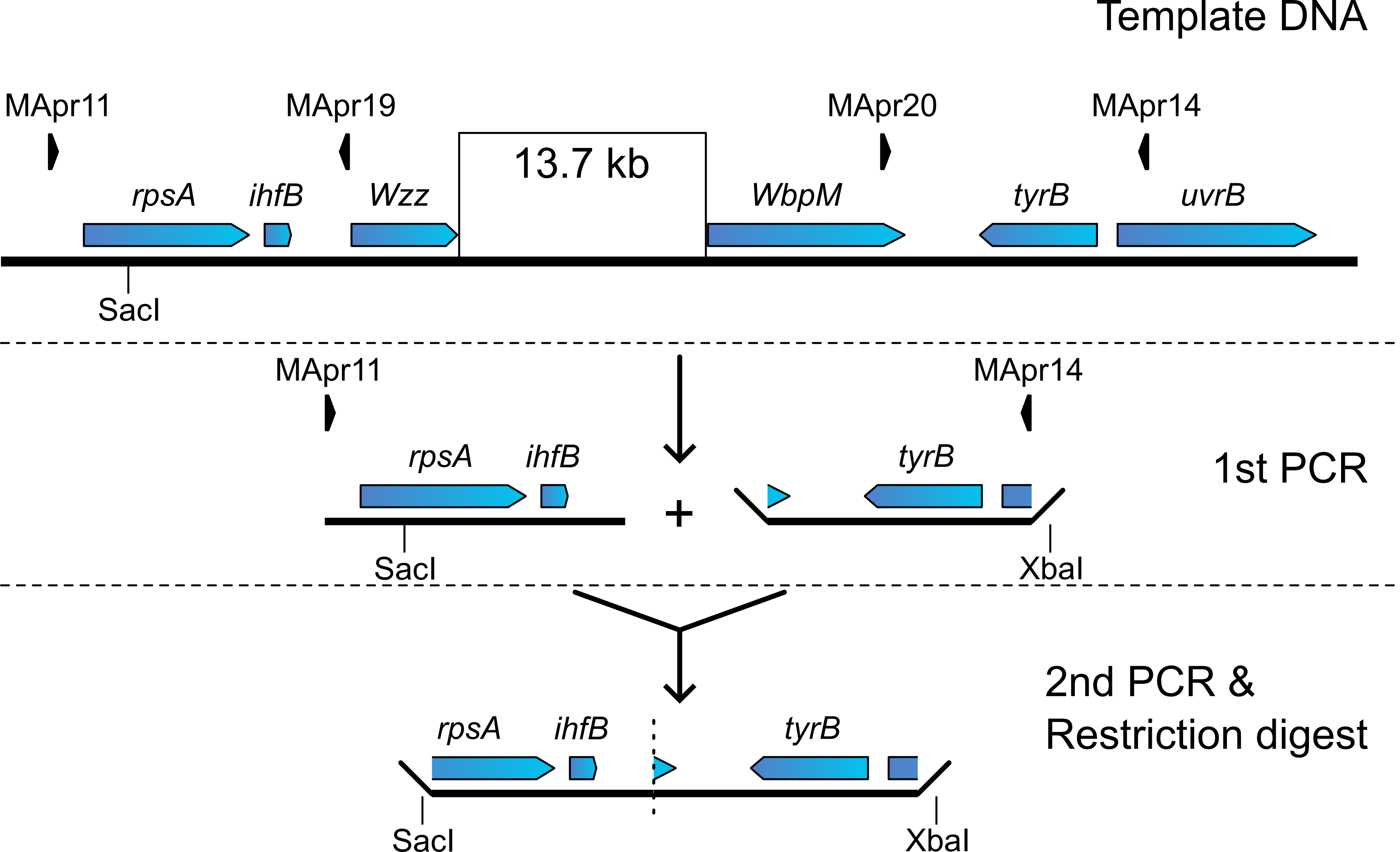

Supplement: S7 Fig — The location of annotated genes is shown as filled blue arrows, primers as filled black arrows, and restriction sites below the horizontal line. The region shown in the top panel represents 25 kb in the PA14 genome and the relative location and size of annotated genes is to scale. DNA (horizontal black line) from a single boiled colony is used as a template for two separate PCR reactions to amplify regions up- and downstream of the OSA cluster. The resulting PCR products are purified and used as template for SOE PCR (2nd PCR round), which produces a 5.6 kb PCR product. The final construct is digested using SacI and XbaI, which enables ligation to allelic replacement vector pNJ1. (TIFF) [file ppat.1012221.s007.tiff]
