# Supplementary material for: Serotype switching in Pseudomonas aeruginosa ST111 enhances adhesion and virulence
Source: PLoS Pathog. 2024 Dec 2;20(12):e1012221. doi: 10.1371/journal.ppat.1012221 (PMC11637443; doi:10.1371/journal.ppat.1012221)
Supplement: S1 Table — Only genes and intergenic regions adjacent to the OSA cluster are compared to their ancestral genome. (DOCX) [file ppat.1012221.s008.docx]

**Table S 1** Comparison of cloned OSA clusters to source genome by BLAST alignment. Only genes and intergenic regions adjacent to the OSA cluster are compared to their ancestral genome.

| **OSA cluster inspection** | **Differences [SNPS/Indels]** | **Genbank accession** |
| --- | --- | --- |
| pMA11O4 | 0 | [OR047832](https://www.ncbi.nlm.nih.gov/nuccore/OR047832.1/) |
| pMA11O12 | 0 | [OR047831](https://www.ncbi.nlm.nih.gov/nuccore/OR047831.1/) |
| pMA11O5 | 0 | [OR453952](https://www.ncbi.nlm.nih.gov/nuccore/OR453952.1/) |
| pMA11O19 | 0 | [OR453956](https://www.ncbi.nlm.nih.gov/nuccore/OR453956.1/) |
| PAO1ΔO+O4 | 0 | [JASSSE000000000](https://www.ncbi.nlm.nih.gov/nuccore/JASSSE000000000) |
| PAO1ΔO+O12 | 0 | [JASSSD000000000](https://www.ncbi.nlm.nih.gov/nuccore/JASSSD000000000) |
| PA14ΔO+O4 | 0 | [JASSSG000000000](https://www.ncbi.nlm.nih.gov/nuccore/JASSSG000000000) |
| PA14ΔO+O12 | 0 | [JASSSI000000000](https://www.ncbi.nlm.nih.gov/nuccore/JASSSI000000000) |
| PA14ΔO+O19 | 0 | [JASSSH000000000](https://www.ncbi.nlm.nih.gov/nuccore/JASSSH000000000) |
| ST111ΔO+O4 | 0 | [JASSSB000000000](https://www.ncbi.nlm.nih.gov/nuccore/JASSSB000000000) |
| ST111ΔO+O12 | 0 | [JASSSA000000000](https://www.ncbi.nlm.nih.gov/nuccore/JASSSA000000000) |
