# Supplementary material for: Serotype switching in Pseudomonas aeruginosa ST111 enhances adhesion and virulence
Source: PLoS Pathog. 2024 Dec 2;20(12):e1012221. doi: 10.1371/journal.ppat.1012221 (PMC11637443; doi:10.1371/journal.ppat.1012221)
Supplement: S2 Table — (DOCX) [file ppat.1012221.s009.docx]

**Table S 2** Comparative genomics between serotype switched engineered strains, analyzed by breseq.

|  | **Genomic differences [SNPS / Indels]** |
| --- | --- |
| ST111ΔO+O4 vs ST111ΔO+O12 | 30/1 |
| PA14ΔO+O4 vs PA14ΔO+O12 | 0/1 |
| PAO1ΔO+O4 vs PAO1ΔO+O12 | 0/0 |
