# Supplementary material for: Serotype switching in Pseudomonas aeruginosa ST111 enhances adhesion and virulence
Source: PLoS Pathog. 2024 Dec 2;20(12):e1012221. doi: 10.1371/journal.ppat.1012221 (PMC11637443; doi:10.1371/journal.ppat.1012221)
Supplement: S3 Table — (DOCX) [file ppat.1012221.s010.docx]

Table S 3 List of primers

| **Primer name** | **Sequence** |
| --- | --- |
| MApr9-pNJ1-fw | AACAAACCCGCGCGATTTAC |
| MApr10-pNJ1-rev | GTAACGCACTGAGAAGCCCT |
| MApr11 OSA HR wzz F | GGACTCCACCGAAATGAGCA |
| MApr14 OSA HR wbpM R | TTATATCTAGAGAACGTCTTGAACTCCCCGT |
| MApr19 OSA HR wzz R | GTAGAGCTTCTCGCCAGGACTACAAACTGATGTCGCCCCA |
| MApr20 OSA HR wbpM F | TGGGGCGACATCAGTTTGTAGTCCTGGCGAGAAGCTCTAC |
| MApr77 wbpM1741 SOE | TGCCAAAGTGCGTCTCAGATGTCCTGGCGAGAAGCTCTAC |
| MApr76 WzzUp502 | ATCTGAGACGCACTTTGGCA |
| MApr35 FW delver OSA | AGAGCTGGATTGTTCTGGAAGC |
| SVpr1 | GTACGAACCGAAAGCCTCCT |
| SVpr3 | AGGAGGCTTTCGGTTCGTACGTCCTGGCGAGAAGCTCTAC |
| MApr30 wzz junc seq | GAGCGGTGAACGAAAGCAAC |
| MApr31 wbpM junc seq | AGCTCAGGTGTTCCTCGTTG |
| MApr79 ihfB148 fw | GCTTTTCCTTGCACTACCGC |
| MApr68 wbpM FW XbaI | TATTATCTAGAACATCGCCATCGAGTTCAGT |
| MApr71 comEA downrev SacI | TAATAGAGCTCATTCAAAGCCCTCGGCACAT |
| MApr74 wbpM369 fw | CCCTGGTGTTCAACTACTGG |
| MApr25 mini-tn7 FW | CGCCTGGGGTAATGACTCTC |
| MApr26 mini-tn7 REV | CTGCCACTCATCGCAGTCTA |
| MApr38 puc18 recomb fw | GAAGCGCGATCACATGGTCCTGCTGGAGTTCGTG  ACCGCCGCCGGGATCACTCTCGGCATGGACGAGC  TGTACAAGTAATCGCCTGGGGTAATGACTCTC |
| MApr39 puc18 recomb rev | TGAAATTGTTATCCGCTCACAATTGAATCTAA  GTATCATTGTTATCCGCTCACAAGTCAACACT  CTTTTTGATAAATTTTCTGCCACTCATCGCAGTCTA |
| MApr41 OSA recomb rev | ATCCGTTCGATCAACTCCGACTTGGTCATGG  TTTTCCCTTCTTTTTCAAGCGGCTAGATCAGCT  CAGGTCTCTTTTAGCACTGCCACTCATCGCAGTCTA |
| MApr56 OSA recomb fw | CGGTGCCATTTCGACGGCAGAAAACAGACTCATTT  TGCGGCTGCTCGGAGTGTGAAGAGAGGAGGGCA  ACGCAACCCGTTCGCCTGGGGTAATGACTCTC |
| MApr61 pUC18x rev | TCCATTGAGTAAGTTTTTAAGCACA |
| MApr73 wbpM fw2 | TCACGTCAACAAGACCCGTT |
| MApr36 pUC18 recver fw | TATTAAAGAGGGGCGTGGGG |
| MApr62 wzz end verif | CGCGGTAGTGCAAGGAAAAG |
| MApr87 mini-ctx recomb rev | TGAAGCTGATGTGCTTAAAAACTTACTCAAT  GGAATTAGCCGTGGCTGGATTCTCACCAA |
| MApr102 mini-ctx recomb fw | TTCATTGATGTTTCCATTCGACCCACTCTCA  GGAGTGAACTTTCTAGGGCGGCGGATTTG |
| MApr108 PA14GI 1f | CGTATTCACGCCCTCGATGA |
| MApr109 PA14GI 1r | TCAATGCGGGTATACGGACG |
| MApr150 PA14pilAfwXbaI | TTAAATCTAGAGGGCTCTTTTCAGCATTA |
| MApr151 PA14pilArevEcoRI | TTAATGAATTCGGAGAGATACATGAAAGC |
