# Supplementary material for: Serotype switching in Pseudomonas aeruginosa ST111 enhances adhesion and virulence
Source: PLoS Pathog. 2024 Dec 2;20(12):e1012221. doi: 10.1371/journal.ppat.1012221 (PMC11637443; doi:10.1371/journal.ppat.1012221)
Supplement: S4 Table — The values were generated from E-test. E. coli ATCC25922 and E. coli NTCT13846 were used for control to COL MIC. E. coli ATCC25922 MIC = 0,250 μg/mL and E. coli NTCT13846 MIC = 4,000 μg/mL. (DOCX) [file ppat.1012221.s011.docx]

**Table S 4** MIC of different antibiotics for P. aeruginosa ST111 and PA14 strains expressing different serotypes. The values were generated from E-test. E. coli ATCC25922 and E. coli NTCT13846 were used for control to COL MIC. E. coli ATCC25922 MIC = 0,250 µg/mL and E. coli NTCT13846 MIC = 4,000 µg/mL.

| **Strain** | **Ciprofloxacin E-test** µg/mL | **Meropenem E-test** µg/mL | **Tobramycin E-test** µg/mL | **Colistin MIC** µg/mL |
| --- | --- | --- | --- | --- |
| PA14 ∆OSA | 0,125 | 0,250 | 2,000 | 1,000 |
| PA14 O4 | 0,064 | 0,250 | 1,000 | 1,000 |
| PA14 O12 | 0,064 | 0,125 | 0,500 | 1,000 |
| PA14 O19 | 0,125 | 0,250 | 0,500 | 1,000 |
| ST111 WT | >32,000 | >32,000 | 64,000 | 0,500 |
| ST111 ∆OSA | >32,000 | >32,000 | 64,000 | 1,000 |
| ST111 O4 | >32,000 | >32,000 | 32,000 | 0,500 |
| ST111 O12 | >32,000 | >32,000 | 32,000 | 1,000 |
