# Supplementary material for: Serotype switching in Pseudomonas aeruginosa ST111 enhances adhesion and virulence
Source: PLoS Pathog. 2024 Dec 2;20(12):e1012221. doi: 10.1371/journal.ppat.1012221 (PMC11637443; doi:10.1371/journal.ppat.1012221)
Supplement: S1 Supplementary Methods — (DOCX) [file ppat.1012221.s012.docx]

Supplementary Methods

**Integrity of cloned OSA clusters and maintenance of genotype after cloning**

To ensure integrity of OSA clusters and strains after cloning, these were sequenced on the Illumina Novaseq 6000 platform and or the Oxford Nanopore flowcell on the MINion device. BLAST alignment was used to inspect OSA clusters, and breseq (0.38.1) (1, 2) was used to detect genomic differences. As breseq requires a completed genome, we could not directly compare genomes of engineered strains, so these were compared to their ancestral wild-type genome (or the closest genome in the case of PAO1 strains) and an in-house script was used to summarize differences (table s2). Integrity of OSA clusters was analyzed by comparison to the O4 cluster (GCF_001420245.1), O12 (GCA_001444975.1), O5 (NZ_CP017149.1), or O19 ([CP127126](https://www.ncbi.nlm.nih.gov/nuccore/CP127126)). While genomic differences were found by mapping reads of engineered strains to their respective reference in PA14 ([CP127126](https://www.ncbi.nlm.nih.gov/nuccore/CP127126)), PAO1 ([NZ_CP017149.1](https://www.ncbi.nlm.nih.gov/nuccore/NZ_CP017149.1)), or ST111 2875 ([CP116727.1](https://www.ncbi.nlm.nih.gov/nuccore/CP116727.1), [CP116728.1](https://www.ncbi.nlm.nih.gov/nuccore/CP116728.1), and [CP116729.1](https://www.ncbi.nlm.nih.gov/nuccore/CP116729.1)) (breseq reports can be found in Supplementary 2 Comparative genomics).

Engineering of PA14ΔO to heterologously express serotype O12 was repeatedly associated with a 26719 bp deletion within genomic island PA14R29 (3) from nucleotide 1300333 to 1327051 (according to the wildtype genome CP127126). Based on gene annotations this genomic region encodes genes associated with the propagation of plasmids *traJ* and *trbJKL*, as well as genes associated with mercury resistance *merRTPADE*. In order to ensure this had no influence on adhesion we went back to construct a new PA14ΔO+O12 strain (PA14ΔO+O12-2) where this deletion had not taken place (verified by WGS and PCR). Comparative genomics analysis with breseq found no genomic differences (SNPS or indels) between PA14ΔO+O12-2 and PA14ΔO+O4, verifying that these are isogenic with the exception of the serotype.

We tested the adhesion of both strains to tissue culture polystyrene (materials and methods) and found no significant difference between their adhesion (Figure S 6). From this, we conclude that the deletion is PA14R29 has no influence on the results presented in this manuscript.

To test if there is an incompatibility between PA14R29 and the serotype O12 cluster, we tested the stability of the genomic island PA14R29 in this new strain by growing overnight cultures and plating these for single colonies (n = 4 cultures). For each culture, we tested 20 colonies by PCR (with primers that are specific to the PA14R29 region: MApr108 and MApr109), found 100% were positive, indicating that the genomic island was intact and that there is no incompatibility between PA14R29, and serotype O12.

**Plasmid construction (allelic replacement vectors)**

**pMA03: Serotype deletion plasmid**

To facilitate deletion of OSA clusters in *P. aeruginosa*, a strain specific allelic replacement vector was constructed using vector pNJ1. Briefly, this was done by amplifying ~2-3 kb fragments up and downstream of the OSA clusters. The resulting PCR products were used as template for SOE PCR to join these fragments together into a “deletion fragment” (Figure S 7). The “deletion fragment” was cut using restriction enzymes and ligated to the allelic replacement vector pNJ1.

For PA14, a boiled colony was used as template for PCR reactions with primers MApr11 & MApr19 to amplify a 2.9 kb region upstream of wzz. A 2.6 kb region downstream of wbpM was amplified by PCR with primers MApr14 & MApr20. The resulting PCR products were diluted and used as a template for PCR reaction with primers MApr14 & MApr11.

In PAO1, primer pair MApr14 & MApr77 was used to PCR amplify a 2.6 kb region downstream of wbpM. A 2.7 kb region upstream of wzz was PCR amplified with primers MApr11 & MApr76. The resulting PCR products were diluted and used as a template for PCR reaction with primers MApr14 & MApr11.

In ST111 2875, primer pair MApr14 & SVpr3 were used to PCR amplify a 2.6 kb fragment downstream of wbpM. A 2.9 kb region downstream of wzz was PCR amplified with primers MApr35 & SVpr1. The resulting PCR products were diluted and used as a template for PCR reaction with primers MApr14 & MApr35.

Deletion fragments and allelic replacement vector pNJ1 were digested using FD XbaI (Thermo Scientific) and FD SacI (Thermo Scientific) according to the manufacturer’s specifications. pNJ1 vector was dephosphorylated using FastAP (Thermo Scientific) according to the manufacturer’s specifications. DNA was quantified by gel electrophoresis and NEB Ligation calculator was used to determine the amount of insert to add to 50 ng plasmid for a 3:1 (insert:plasmid) ratio. Inserts and plasmids were mixed 3:1 and ligated using T4 Ligase (Thermo Scientific) according to manufacturer’s specifications. 5 µl ligation mix was used to transform competent *E. coli* cc118λpir cells. Plasmid was verified by PCR and Sanger sequencing using primers MApr9, MApr10, MApr30, MApr31, and MApr79.

**pMA10: Serotype tagging plasmid**

To facilitate tagging of OSA clusters in *P. aeruginosa*, a strain specific allelic replacement vector was constructed using vector pNJ1. Briefly, this was done by amplifying ~1 kb fragments of the OSA clusters. The PCR fragment was cut using restriction enzymes and ligated to the allelic replacement vector pNJ1. Briefly, in each strain background, a single colony was boiled and used as template for PCR using primers MApr68 & MApr71 was digested using FD XbaI (Thermo Scientific) and FD SacI (Thermo Scientific), according to manufacturer’s specifications. Purified pNJ1 plasmid was similarly digested with FD XbaI (Thermo Scientific), FD SacI (Thermo Scientific), and treated with FastAP (Thermo Scientific). Purified DNA was quantified by gel electrophoresis, mixed (3:1, insert:plasmid) to 50 ng plasmid (calculated using NEB Ligation calculator) and ligated using T4 ligase (Thermo Scientific) according to manufacturer’s specifications. Plasmids were verified by PCR and Sanger sequencing using primers MApr9 & MApr10. Correct integration into *P. aeruginosa* was verified by PCR with primers MApr10 & MApr74 by a 2.4 kb band.

**Plasmid construction (recombineering)**

**pMA09: Preparation of recombineering tn7 vector**

Plasmid AKN69 was purified using the plasmid purification kit (Macherey-Nagel) according to manufacturer’s specifications for low copy plasmids. The plasmid was digested with FD XhoI (Thermo Scientific) and FD NcoI (Thermo Scientific), run on gel electrophoresis and band at 1500 bp were cut and purified using the PCR clean up kit (Macherey-Nagel). Recipient vector pUC18R6kT-mini-Tn7T-Km was prepared and linearized by PCR with primers MApr38 & MApr39 and gel purified. 500 ng pUC18 PCR fragment and digested AKN69 fragment was electroporated into competent GBdir-pir116 cells and grown on LB+Km35 plates. Correct integration was verified by fluorescence microscopy of colonies.

**pMA11 Serotype switch plasmid construction**

Plasmid pMA09 was linearized by PCR using primers MApr41 & MApr56 (PAGE purified primers), and purified with the PCR clean-up kit (Macherey-Nagel). PCR product was then digested with DpnI and gel purified. Genomic DNA from serotype donor strains was purified from 1 ml overnight culture with the Monarch HMW DNA Extraction Kit for Tissue (NEB) and verified by gel electrophoresis. Depending on the serotype donor strain, 85 µl gDNA was digested with RsrII (for O4), Sfil (O12 & O5), or MauBI & SgrDI (O19) for up to 1 hour. Double digestion for serotype O19 gDNA was done in sequence without purification steps in between. Digested gDNA was purified by ethanol precipitation, eluted in 30 µl MQ H_2_O overnight at RT and verified by gel electrophoresis. 5-10 µl digested gDNA was used together with linearized pMA09 to electroporate RecET induced GBdir-pir116 competent cells. Correct recombineering was selected by plating these on LB+tet8+km35. Plasmids were verified by PCR using primer pairs MApr9 & MApr61, MApr10 & MApr73, and MApr36 & MApr62. Colonies positive for all PCR reactions were sequenced by Sanger and whole-genome sequencing and used for transformation of *P. aeruginosa*.

**mini-ctx2 msfGFP aacC1**

Performed PCR on pBG42-msfGFP (4) with primers MApr87+MApr102, purified PCR product. Digested mini-ctx2 with FD SacI and subsequently purified digest. Digested PCR product and plasmid were electroporated into competent GBdir-pir cells (rescued for 1h in LB at 37) and plated on LB+tet8+Gm15. Transformants were verified by PCR and fluorescence microscopy.

**mini-ctx2 mKate aacC1**

Performed PCR on pBG42-mKate (Hansen, M, unpublished) with primers MApr87+MApr102, purified PCR product. Digested mini-ctx2 with FD SacI and subsequently purified digest. Digested PCR product and plasmid were electroporated into competent GBdir-pir cells (rescued for 1h in LB at 37) and plated on LB+tet8+Gm15. Transformants were verified by PCR and fluorescence microscopy.

# References

1. Barrick JE, Colburn G, Deatherage DE, Traverse CC, Strand MD, Borges JJ, Knoester DB, Reba A, Meyer AG. 2014. Identifying structural variation in haploid microbial genomes from short-read resequencing data using breseq. BMC Genomics 15:1039.

2. Deatherage DE, Barrick JE. 2014. Identification of Mutations in Laboratory-Evolved Microbes from Next-Generation Sequencing Data Using breseq, p. 165–188. *In* Sun, L, Shou, W (eds.), Engineering and Analyzing Multicellular Systems: Methods and Protocols. Springer, New York, NY.

3. Lee DG, Urbach JM, Wu G, Liberati NT, Feinbaum RL, Miyata S, Diggins LT, He J, Saucier M, Déziel E, Friedman L, Li L, Grills G, Montgomery K, Kucherlapati R, Rahme LG, Ausubel FM. 2006. Genomic analysis reveals that Pseudomonas aeruginosa virulence is combinatorial. Genome Biology 7:1–14.

4. Zobel S, Benedetti I, Eisenbach L, de Lorenzo V, Wierckx N, Blank LM. 2015. Tn7-Based Device for Calibrated Heterologous Gene Expression in Pseudomonas putida. ACS Synth Biol 4:1341–1351.
